# Gas7-Deficient Mouse Reveals Roles in Motor Function and Muscle Fiber Composition during Aging

**DOI:** 10.1371/journal.pone.0037702

**Published:** 2012-05-25

**Authors:** Bo-Tsang Huang, Pu-Yuan Chang, Ching-Hua Su, Chuck C.-K. Chao, Sue Lin-Chao

**Affiliations:** 1 Institute of Molecular Biology, Academia Sinica, Taipei, Taiwan; 2 Graduate Institute of Medical Sciences, College of Medicine, Taipei Medical University, Taipei, Taiwan; 3 Department of Biochemistry and Molecular Biology, Chang-Gung University, Taoyuan, Taiwan; Institut de Génomique Fonctionnelle, France

## Abstract

**Background:**

Growth arrest-specific gene 7 (Gas7) has previously been shown to be involved in neurite outgrowth *in vitro*; however, its actual role has yet to be determined. To investigate the physiological function of Gas7 *in vivo*, here we generated a Gas7-deficient mouse strain with a labile Gas7 mutant protein whose functions are similar to wild-type Gas7.

**Methodology/Principal Findings:**

Our data show that aged Gas7-deficient mice have motor activity defects due to decreases in the number of spinal motor neurons and in muscle strength, of which the latter may be caused by changes in muscle fiber composition as shown in the soleus. In cross sections of the soleus of Gas7-deficient mice, gross morphological features and levels of myosin heavy chain I (MHC I) and MHC II markers revealed significantly fewer fast fibers. In addition, we found that nerve terminal sprouting, which may be associated with slow and fast muscle fiber composition, was considerably reduced at neuromuscular junctions (NMJ) during aging.

**Conclusions/Significance:**

These findings indicate that Gas7 is involved in motor neuron function associated with muscle strength maintenance.

## Introduction

*Growth arrest-specific gene 7* (*Gas7*) was discovered in growth-arrested NIH3T3 fibroblasts using a retrovirus-based gene search strategy [1], [2]. Previous studies have shown that Gas7 is expressed in the central nervous system (CNS) including the cerebral cortex, hippocampus, and cerebellum [3]. Additionally, impeding endogenous Gas7 in primary cultures of hippocampal cells, cerebellar Purkinje neurons, and NGF stimulated-PC12 cells has shown that Gas7 is required for neurite outgrowth [3], [4]. The Gas7 protein interacts directly with F-actin to enhance actin polymerization through interaction with N-WASP [5], [6], and Gas7 contains both WW and F-BAR domains, which have been reported to be associated with membrane protrusions and neurite outgrowth [7], [8]. Recent *in vitro* studies have reported a novel role for Gas7 in maintaining microtubule stability and polymerization with the Gas7/Tau complex [9], [10].

To investigate the function of Gas7 *in vivo*, we established a Gas7-deficient mouse model by replacing Gas7 with a labile mutant protein. The Gas7-deficient mice had normal brain morphology but exhibited motor dysfunction as evidenced by rotarod performance. The rotarod is a widely used behavioral test for measuring motor coordination, balance, and motor activity, all of which involve neuronal processing for skeletal muscle control by the brain and the spinal cord [11]–[13]. Therefore, our findings led us to examine potential mechanisms by which Gas7 deficiency leads to pathogenic changes in spinal motor neurons and skeletal muscle. Axons of spinal motor neurons innervate the muscle fibers and form the neuromuscular junctions (NMJs), synaptic connections where acetylcholine (ACh) is released into nicotinic acetylcholine receptors (nAChR) to induce muscle contraction [14]–[16]. Some studies have indicated that loss of motor neuron function can cause skeletal muscle atrophy or abnormal muscle fiber composition, resulting in diseases such as amyotrophic lateral sclerosis (ALS), spinal muscular atrophy (SMA), or Charcot-Marie-Tooth (CMT) disease type 1A [17]–[19].

Skeletal muscle is stimulated to contract by the release of ACh from the axon terminal of motor neurons, which diffuses across the narrow synaptic cleft and binds to nAChR clustered in the postsynaptic muscle membrane [20], [21]. Other contractile properties of skeletal muscle depend on the different composition of muscle fibers identified by myosin heavy chain (MHC) isoforms; fibers containing fast MHC isoforms have faster maximal shortening velocity than fibers with the MHC I (slow/β cardiac) isoform [15], [22]. In aging animals and humans, a complicated interplay of factors including altered hormonal profiles, exercise, changes in neuromuscular activity, and decreased motor neuron axon innervation causes a gradual loss of muscle strength [23]–[25]. However, motor neuron terminal sprouting is one important mechanism that helps compensate for loss of strength during aging or motor neuron disease [26].

In the present study, we detail the creation and characterization of the Gas7-deficient mouse that expresses a labile Gas7 mutant protein that produces a similar phenotype to wild-type Gas7. Through extensive behavioral testing, we found that aged Gas7-deficient mice have motor activity defects. These defects may be associated with fewer motor neurons and muscle weakness but not with a loss of balance. Following a detailed analysis, we found abnormal muscle fiber composition and reduction in motor neuron terminal sprouting in aged Gas7-deficient mice, which have mild Gas7 expression in muscle fibers and the presynaptic region of the NMJ. This work reveals that Gas7 may play a role in motor neuron function and regulation of muscle fiber composition.

## Results

### Generation and Characterization of Gas7-deficient Mice

**Figure 1:**
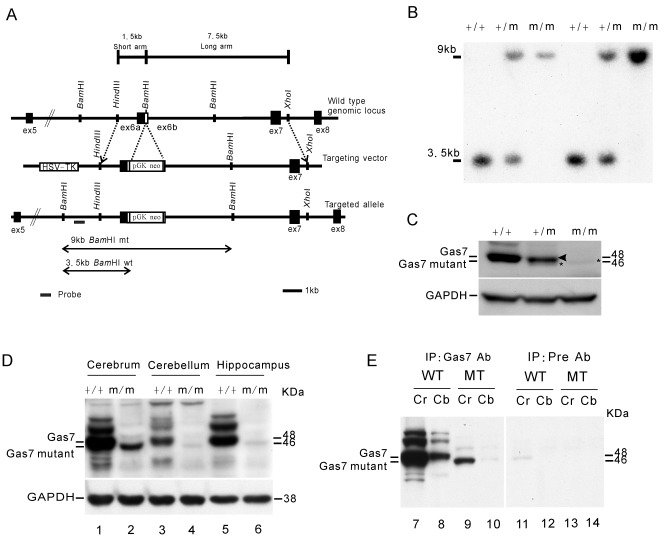
Disruption of *gas7* gene and confirmation of Gas7 mutant protein expression. (A) Maps of the *Gas7* wild-type (+/+) and mutant (m/m) alleles. The *gas7* gene targeting vector contained a *neo* cassette inserted into the unique *Bam*HI site in exon 6 b. (B) Southern blot analysis of genomic DNA from *gas7* ^+/+^, *gas7* ^+/m^ or *gas7* ^m/m^ mice. Genomic DNA samples prepared from mouse tail were digested by *Bam*HI and hybridized with a probe specific to the region between exon 5 and 6 of the *Gas7* gene (bar) to distinguish the wild-type alleles from mutants. The band at 3.5 kb indicates the wild-type allele harboring the *Bam*HI site. The 9 kb band represents the mutant allele, which was resistant to *Bam*HI digestion. (C) Expression of *Gas7* was examined in brain lysates from *gas7* ^+/+^, *gas7* ^+/m^ or *gas7* ^m/m^ mice. Gas7-deficient mice generated a mutant protein (asterisk) smaller in size than the wild-type Gas7 (arrowhead). (D) Expression of Gas7 and Gas7 mutant were examined in three brain regions including cerebral cortex, cerebellum, and hippocampus of wild-type and deficient mice. (E) Gas7 mutant expression in the cerebrum and cerebellum of the Gas7-deficient mice was confirmed by IP-Western using anti-Gas7 antibody. Gas7 mutant protein was not detected by pre-immune antibody in the cerebrum or cerebellum of the Gas7-deficient mice.

To study the function of the Gas7 protein *in vivo*, we constructed a targeting vector to disrupt the *Gas7* gene by inserting a Neo-loxP cassette with a stop codon into the *Bam*HI site in exon 6 b of the *Gas7* genomic locus. With a 1.5 kb short arm and a 7.5 kb long arm flanking the Neo-loxP cassette, the targeted allele was predicted to produce a 9 kb fragment after *Bam*HI digestion using a *gas7*-specific probe, thus distinguishing the targeted from the wild-type allele (Figure 1A). The genotypes of progeny mice were examined by Southern blot analysis of genomic DNA prepared from tail tissues (Figure 1B), and the expected *Bam*HI-digested genomic DNA fragments for the wild-type (+/+, 3.5 kb), heterozygous (+/m, both 3.5 and 9 kb), and homozygous (m/m, 9 kb only) genotypes were found.

**Figure 2:**
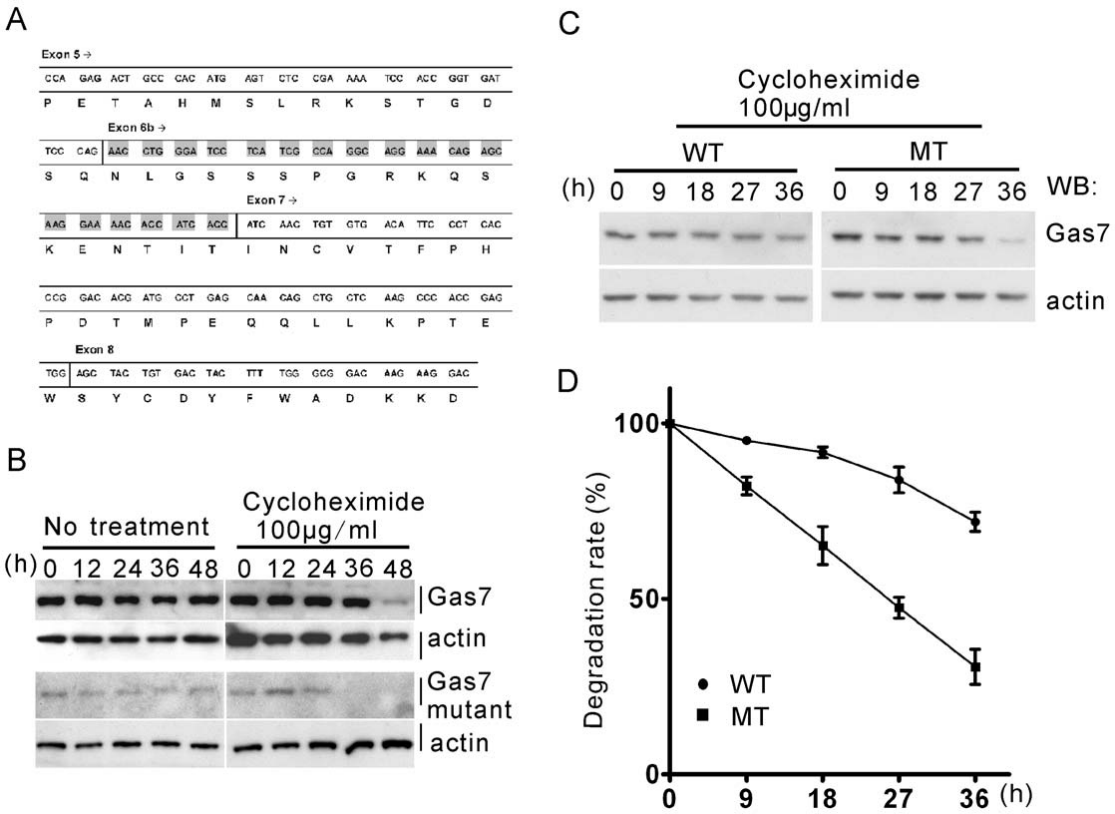
The truncated sequence of the Gas7 mutant is associated with control of Gas7 protein stability. (A) Total RNA from the cerebrum of Gas7-deficient mice was subjected to RT-PCR followed by direct cloning of *gas7* cDNA. Sequence analysis showed that a total length of 54 nucleotides (comprising exon 6 b) was deleted from the *Gas7* wild-type allele. (B) The endogenous Gas7 mutant protein degraded faster than wild-type Gas7 in primary culture of cortical neurons. A Primary culture of cortical neurons was isolated at E16.5 from Gas7 wild-type or deficient mice. At DIV7, protein stability was analyzed with or without 100 µg/ml cycloheximide at the indicated time points. (C) The ectopically expressed Gas7 mutant protein degraded faster than wild-type Gas7 in 293 T cells. Overexpression of Gas7 wild-type and mutant protein in transiently transfected 293 T cells was analyzed by Western blot. 293 T cells were transfected with 0.5 µg Gas7 wild-type (WT) or 2 µg mutant (MT) plasmids, followed by culturing in the presence of 100 µg/ml cycloheximide, and harvesting at 0, 9, 18, 27, and 36 h after treatment. (D) The degradation rate of the Gas7 mutant protein is about 50% while the wild-type is about 15% at 27 hours. The results reflect the mean of triplicate experiments.

Previous studies have demonstrated that Gas7 is expressed abundantly in brain tissue [3]. To see whether Gas7 protein was absent in homozygous Gas7-deficient mice, Gas7 protein levels in whole brain lysates from the three different genotypes of mice were examined by Western blotting (Figure 1C). Gas7 protein was detected at 48 kDa in both wild-type and heterozygous mice; however, an additional weak band with an approximate molecular weight of 46 kDa was unexpectedly found in both heterozygous and homozygous Gas7-deficient mice. In a more detailed study aimed at localization of the protein in different brain subregions, the 46 kDa band was again detected in the cortex, hippocampus and cerebellum of Gas7-deficient mice, though predominantly in the cortex (Figure 1D, lanes 2, 4, and 6). Immunoprecipitation (IP) using Gas7 antibodies was then used to confirm that the 46 kDa protein was indeed created by the *gas7* targeting allele (Figure 1E). The 46 kDa protein was pulled down from both cerebral and cerebellar lysates of Gas7-deficient mice by anti-Gas7 antibody. Moreover, RT-PCR and sequencing analysis showed that the truncated region, a fragment of 54 bp encoding 18 amino acids without any functional domain or motif, was located at exon 6 b of *Gas7* (Figure 2A). With this truncated mRNA, a Gas7 mutant of about 46 kDa was generated by the Gas7-deficient mice. Furthermore, we observed that the level of the Gas7 mutant protein is significantly reduced in the deficient mice (Figure 1C and D). Protein degradation assay with cycloheximide revealed that the endogenously expressed Gas7 mutant protein is more easily degraded than the wild-type in primary cortical cultured neurons from E16.5 wild-type and Gas7-deficient mice (Figure 2B). However, to ensure the protein stability of the mutant Gas7, we ectopically over-expressed wild-type and mutant Gas7 in 293 T cells and harvested at the indicated time points. We quantified the degradation rate of the mutant protein to be about 50% and wild-type about 15% at 27 hours (Figure 2C and D). Here, we demonstrated that the Gas7 mutant protein with the truncated region has a shortened protein half-life compared to wild-type protein. Therefore, our mouse model is a Gas7-deficient rather than a conventional Gas7 knockout, and this truncated form of Gas7 was named Gas7 mutant protein.

### Gas7-deficient Mice Correspond to a Mild Gas7 Protein Expression Model

**Figure 3:**
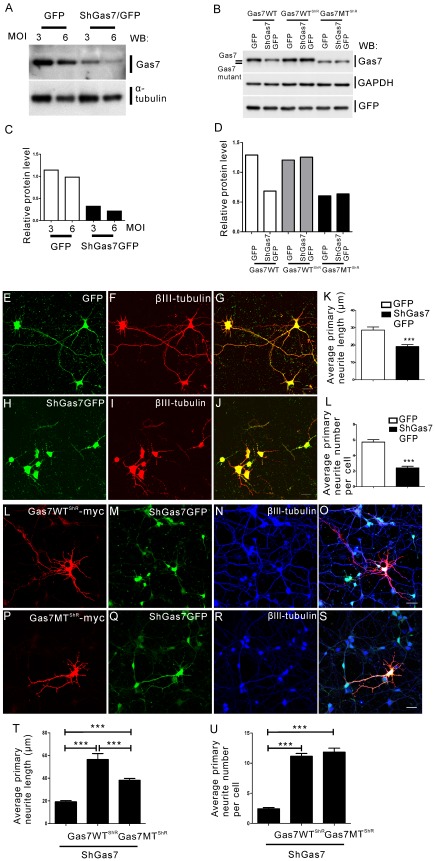
Comparison of the rescue ability of Gas7 wild-type and mutant protein. GFP and shGas7GFP were inserted into pLKO.1-puro plasmid for production of lentiviral particles. The shGas7GFP specifically targets the sequence 5′-^1081^GTG GAA ATG ATC CGA CAA CAT^1101^-3′ located on the open reading frame of *gas7* to knock down Gas7 expression. (A and C) Western blot showing that endogenous Gas7 efficiency was knocked down by shGas7GFP overexpression in cortical neuron primary culture. The relative protein level of Gas7 was normalized to α-tubulin. (B) Gas7WT^ShR^ and Gas7MT^ShR^ exhibit resistance to Gas7 shRNA knock down. All plasmids were transiently transfected (0.5 µg) into 293 T cells. Western blot detected Gas7, GAPDH, and GFP. The relative protein level of Gas7 was normalized to GAPDH. (E-G) Normal morphology of primary cortical neurons following overexpression of GFP. (H–J) shGas7GFP overexpression induced disruption of neurite outgrowth of primary cortical neurons. (F) and (I) Distribution of βIII-tubulin (red), a neuronal marker. (G) and (J) Merged GFP-βIII-tubulin and shGas7GFP-βIII-tubulin signals, respectively. Scale bar, 20 µm. (K) and (L) Bar graph of the average length (K) and number (L) of primary neurites in cortical neurons with shGas7GFP expression is significantly decreased as compared with GFP expression. (30 cells were calculated in each group). Data points represent mean ± SEM, ***P<0.001. (L–S) Representative images of cortical neurons with shGas7GFP and Gas7WT^ShR^-myc co-transfection (L–O) or shGas7GFP and Gas7MT^ShR^-myc co-transfection (P–S). (T) and (U) Bar graph of the average length (T) and number (U) of primary neurites in cortical neurons expressing shGas7GFP, shGas7GFP with Gas7WT^ShR^-myc, or Gas7MT^ShR^-myc expression. Data points represent mean ± SEM, ***P<0.001. No significant difference in the average number of primary neurites per cortical neuron was observed between shGas7GFP co-transfected with Gas7WT^ShR^-myc or Gas7MT^ShR^-myc. (25 cells were calculated in each group). Images in (L) and (P) were obtained by sequential staining with anti-myc antibody and Cy3 secondary antibody (red). (M) and (Q) show GFP expression (green). (N) and (R) show βIII-tubulin staining with anti-βIII-tubulin antibody followed by incubation with Cy5 secondary antibody (blue). Scale bar, 20 µm.

Having shown that the Gas7 mutant protein is less stable than wild-type, we next attempted to ascertain whether its function is the same as wild-type or whether the protein is a loss- or gain-of-function mutant. To distinguish between these possibilities, we performed knockdown and rescue assays. We constructed a lentiviral vector containing coding regions for a small hairpin RNA (shRNA) specific for Gas7 to down regulate Gas7 expression and green fluorescent protein (GFP) to identify transfected cells. To assess the degree of suppression, we infected DIV2 primary cortical neurons from the cortex of E16.5 mice with lentivirus particles with shGas7GFP. After 48 hours of infection, cells were harvested and Gas7 expression was quantified by Western blotting (Figure 3A). In figure 3C, the histogram of relative protein level indicates that the endogenous Gas7 protein was reduced about 80% by the lentiviral vector carrying shGas7GFP as compared with control GFP. To examine whether Gas7 suppression affected neurite outgrowth of primary cortical neurons, we used an immunofluorescent antibody against βIII-tubulin, a specific neuron marker for neurite morphological analysis, followed by detection of GFP fluorescence. We observed that the neurite formation of primary cortical neurons in cells infected with shGas7GFP had obvious defects whereas those exposed only to control GFP grew normally (compare Figure 3H–J and E–G, respectively). The histograms correspond to significantly decreased average primary neurite length and number in shGas7GFP transfected primary cortical neurons as compared with control GFP (Figure 3K and L). This result confirmed that the Gas7 shRNA specifically reduced endogenous Gas7 in primary cortical neurons, resulting in neurite outgrowth defects.

We next examined whether the deficiency in neurite outgrowth could be rescued by expressing wild-type or mutant Gas7 protein. We constructed Gas7 wild-type and mutant shRNA-resistant plasmids, Gas7WT^ShR^ and Gas7MT^ShR^, that contained two silent nucleotide substitutions targeting a sequence of shGas7. 293 T cells were transfected with Gas7 wild-type, Gas7WT^ShR^ and Gas7MT^ShR^ plasmids individually with control GFP or shGas7GFP. After a media change and 24 hours incubation, cells were harvested and prepared cell lysate was assayed for Gas7 protein by Western blot (Figure 3B). The histogram of relative protein level showed Gas7WT^ShR^ and Gas7MT^ShR^ were effectively resistant to Gas7 shRNA (Figure 3D). We then tested whether expression of the Gas7WT^ShR^ and Gas7MT^ShR^ rescued the neurite defects of primary cortical neurons induced by Gas7 shRNA. The images and average primary neurite length and number showed that expression of Gas7WT^ShR^ or Gas7MT^ShR^ in the presence of shGas7GFP permitted primary cortical neurons to increase neurite outgrowth (Figure 3L–O and P–S, T and U). The average length and number of primary neurites were identified by myc staining with GFP expression (Figure 3L, M and P, Q) combined with βIII-tubulin staining (Figure 3N, O and R, S; Figure S1K–N). In primary cortical neurons carrying control GFP, overexpression of Gas7WT^ShR^ or Gas7MT^ShR^ also demonstrated that both wild-type and mutant Gas7 protein have the ability to promote primary neurite length and number (Figure S1A–D, E–H, I and J). These results indicate the Gas7 mutant protein exhibits a rescue ability similar to the wild-type protein. Together with the results shown in figure 2B, C, and D, these data reveal that a low level of Gas7 protein is sufficient for initiation of neurite outgrowth; however, a sustained level of Gas7 is required for regular neurite elongation. These data indicate that Gas7-deficient mice correspond to a mild Gas7 protein expression model useful for further characterization and investigation of the biological function of Gas7.

### Impaired Motor Activity in Gas7-deficient Mice During Aging

**Figure 4:**
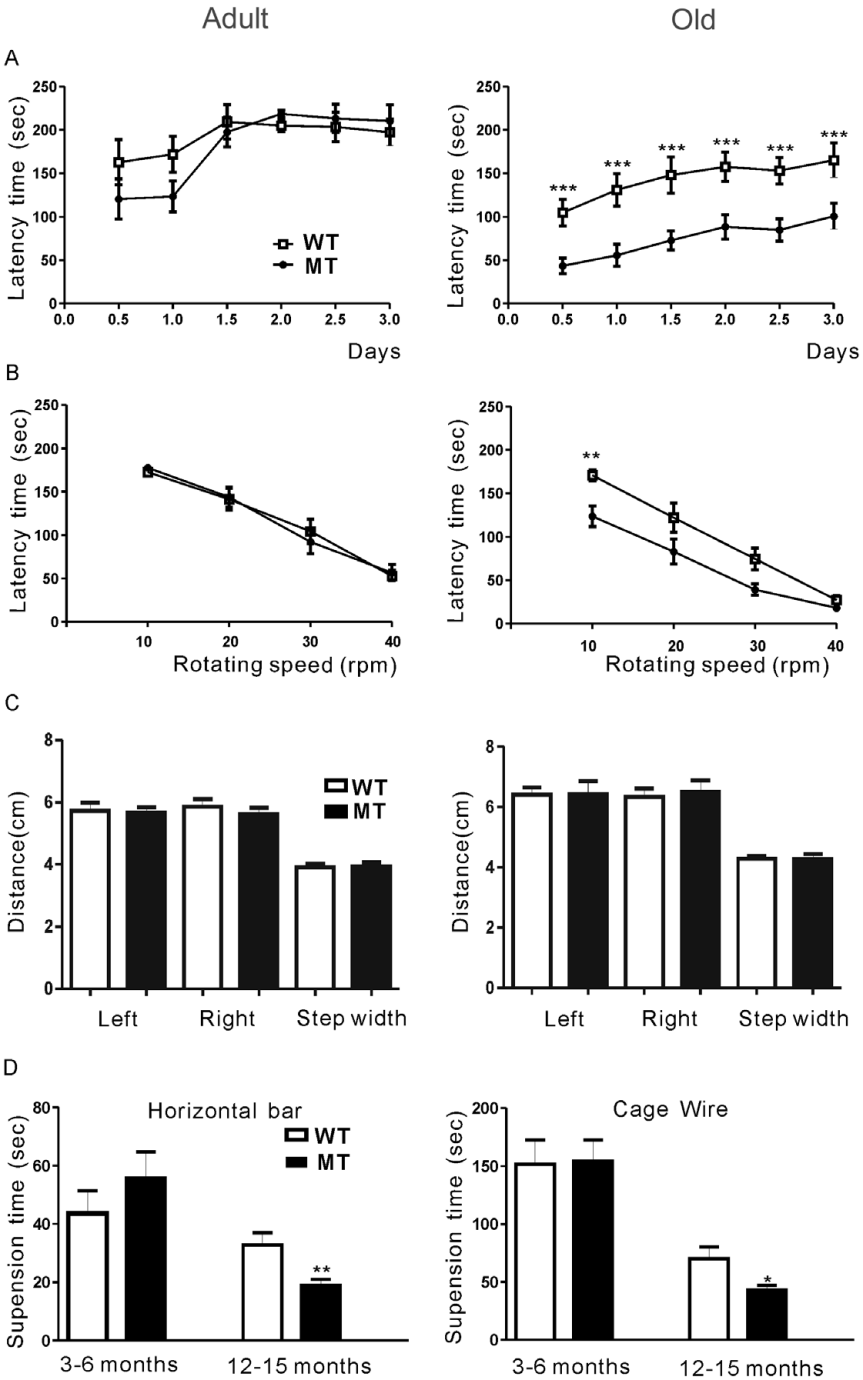
Motor activity defects in old Gas7-deficient mice. Wild-type (WT) and Gas7-deficient (MT) mice were classified into adult (3 to 6 months) and old (12 to 15 months) groups. (A) Rotarod test of wild-type and Gas7-deficient mice. The latency time indicates how long mice were able to walk on an accelerating rotarod at speeds from 5 to 40 rpm. No significant difference between adult wild-type and Gas7-deficient mice was observed (n  = 22 each). In contrast, the time before falling decreased significantly for old Gas7-deficient mice compared to old wild-type mice (n  = 15 each). Data points are mean ± SEM, ***P<0.001. (B) Adult Gas7-deficient mice performed no differently in regards to latency time than wild-type mice in four independent rotarod tests performed at constant speed (n  = 22) (left panel). By contrast, old Gas7-deficient mice were significantly less able than old wild-type mice to stay on the rod, especially at lower rotation speeds with higher latency (mean ± SEM, n  = 15, ** *P*<0.01) (right panel). (C) There was no difference between wild-type and Gas7-deficient mice in both adult (n  = 22) and old (n  = 15) groups in either stride length or width. Effects on gait were examined by analysis of footprints made with waterproof black ink on white paper. Mice with ink applied to the hind paws were induced to walk through a dark tube (43 cm × 7 cm × 10 cm). The step length (left-to-left and right-to-right distance) and width (left-to-right distance) were measured from their footprints. (D) Hanging tests to measure muscle strength. In the old group, the hanging ability decreased significantly in the Gas7-deficient mice compared to the wild-type (n  = 15 each); however, adult mice showed no difference between groups (n  = 22 in each group). Mean ± SEM, **P<0.01, *P<0.05.

In general, Gas7-deficient mice are viable and fertile and appear to develop and breed normally (Table S1). Previous immunohistochemical results had demonstrated an abundant expression of Gas7 in the cortex, hippocampus, and cerebellum; furthermore, it had been shown that Gas7 was associated with neurite outgrowth in Purkinje neurons [3]. We therefore examined the expression of Gas7 in wild-type and deficient mouse brains by immunofluorescence. Expression of Gas7 in the brains of Gas7-deficient mice was markedly decreased without gross alterations in neuronal morphology (Figure S2). However, our previous study showed that Gas7 is involved in neurite outgrowth of Purkinje neurons in cerebellum, which is known as the brain region responsible for motor activity [27]. At present, rotarod performance is one general test to measure motor activity in mice [28], [29]. In adult mice, a rotarod assay revealed no difference in latency time between Gas7 wild-type and Gas7-deficient mice at either accelerating speed or constant velocity (Figure 4A and B, left panels). By contrast, in the aged group, Gas7-deficient mice performed significantly worse than wild-type mice in both tests (Figure 4A and B, right panels). The decreased ability of old Gas7-deficient mice to stay on the rotarod suggests that they may have motor activity defects correlated with limb control or loss of balance [30]–[32]. As assessed by stride analysis for balance detection, the Gas7-deficient mice did not show signs of ataxia or affected walking gait (Figure 4C). We further examined muscle strength by carrying out a hanging test and observed significant differences in strength between Gas7-deficient mice and their wild-type littermates in the aged mice that were not seen in the adult group (Figure 4D). These tests revealed that Gas7-deficient mice display significant motor activity defects associated with loss of muscle strength during aging.

### Aged Gas7-deficient Mice Exhibit Fewer Spinal Cord Motor Neurons

**Figure 5:**
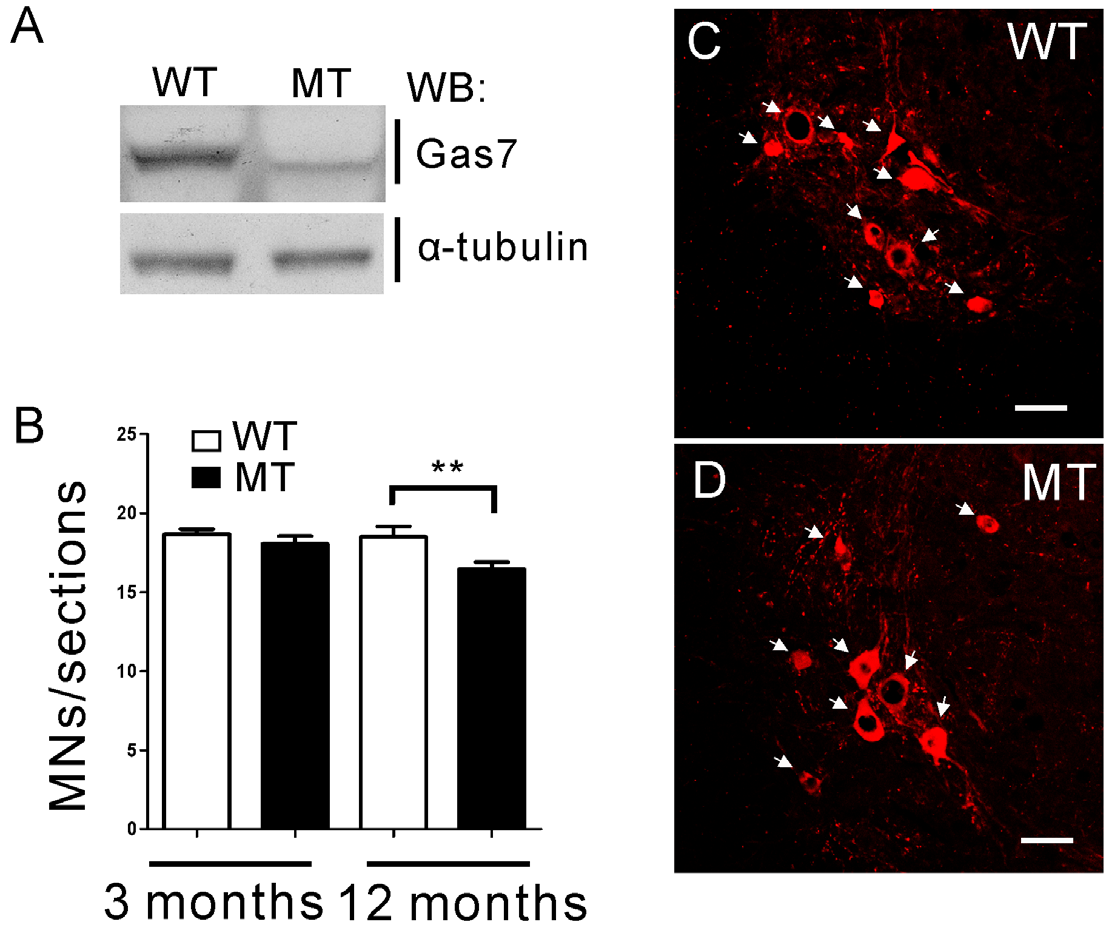
The number of motor neurons is decreased in old Gas7-deficient mice. (A) Western blot showing endogenous Gas7 wild-type and mutant protein expression in the spinal cord of 12 months old wild-type (WT) and Gas7-deficient (MT) mice. (B) The average number of motor neurons per lumbar spinal cord section in adult and old wild-type (WT) and Gas7-deficient (MT) mice were determined from 50 sections each from three mice. Mean ± SEM, ***P<0.001. (C) and (D) ChAT staining of coronal sections of lumbar spinal cords showing motor neurons of ventral horn in Gas7 wild-type (WT) and deficient (MT) mice at 12 months of age. Cy3-conjugated ChAT antibody (red). The ChAT stained motor neurons were indicated by white arrows. Scale bar is 50 µm.

In order to determine the cause of motor activity defects in old Gas7-deficient mice, we focused on the analysis of spinal motor neurons and the skeletal muscle system. Several physiological and pathological studies have shown that motor neurons of the spinal cord are important for triggering skeletal muscle contraction that affects motor coordination [16], [33]–[36]. First, we confirmed that the expression pattern of the Gas7 mutant protein is the same in the spinal cord as in the cortex, hippocampus and cerebellum (Figure 5A). Second, we analyzed motor neurons of the ventral horn in the spinal cord, which respond to skeletal muscle force generation to stimulate movement [37], in Gas7 wild-type and deficient mice by choline acetyltransferase (ChAT) staining (Figure 5C and D). The average number of motor neurons in the ventral horn of 12-month-old Gas7-deficient mice was decreased compared to wild-type mice; by contrast, no significant difference was observed in the analogous 3-month-old groups (Figure 5B). Thus, the reduction in the number of motor neurons of the spinal cord in aged Gas7-deficient mice may be one factor causing motor activity defects.

### Gas7 Localizes at the Presynaptic Region of the NMJ that is Correlated with Axon Terminal Sprouting

**Figure 6:**
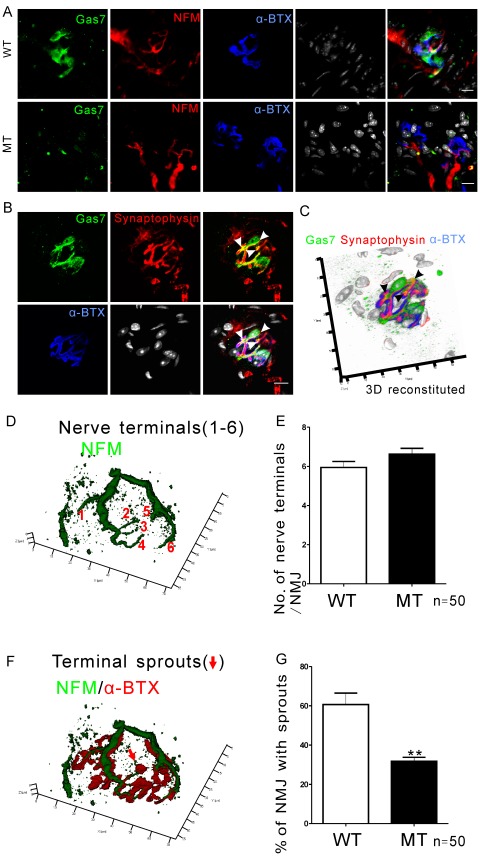
Gas7 localizes at the presynaptic region of the NMJ and is associated with axon terminal sprouting. (A) Gas7 aggregates at the axon terminal region of NMJ in wild-type (WT) soleus. Only weak Gas7 expression was detected in the NMJs of the soleus from the Gas7-deficient (MT) mice. (B) Gas7 co-localizes with synaptophysin partially. Images show separate and merged signals of Gas7, synaptophysin, nAChR cluster, and nuclei. FITC-conjugated Gas7 antibody was used to visualize Gas7 localization (green); Cy5-conjugated NFM antibody indicates neurites of axon terminals (red) or Cy5-conjugated synaptophysin indicates the presynaptic vesicles (red); Cy3-conjugated α-BTX was used to identify nAChR cluster (blue), and nuclei were visualized using Hoechst stain. White arrows mark co-localization areas. Scale bar, 10 µm. (C) The 3-D construction was obtained from 13 slides. It shows that Gas7 co-localizes with synaptophysin in the NMJ (black arrows). The resolution in the z-direction in this image is 1 µm. (D) and (F) The shape of the NMJ and the number of branches of the axon terminal were determined by immunofluorescence. The presence of small nAChR clusters containing nerve termini can be observed at the end of a sprout (red arrow). NFM staining was used to determine nerve axons (green). nAChRs were stained with α-BTX (red). (E) The number of major nerve terminal branches (numbered 1–6 in D) per NMJ did not differ significantly in old wild-type (WT) and Gas7-deficient (MT) mice. (G) Compared to wild-type (WT) mice, the percentage of NMJs exhibiting terminal sprouts was significantly lower in Gas7-deficient (MT) mice (n  = 3 mice in each group, 50 NMJs). Mean ± SEM, **P<0.01.

After we identified spinal motor neuron defects in the old Gas7-deficient mice, we examined neuromuscular junctions (NMJs), which are another important connection system between axon terminals of spinal cord motor neurons and skeletal muscle that function to release neurotransmitter to postsynaptic receptors, thus triggering muscle contraction [14], [16]. Each NMJ consists of a presynaptic nerve terminal of an α-motor neuron arising in the spinal cord, the enveloping Schwann cells, and the muscle end-plate, a postsynaptic structure consisting predominantly of nAChRs [14]. The NMJs in soleus muscles of 12-month-old mice were examined by confocal immunofluorescence imaging after incubation with fluorescently labelled antibodies to Gas7, to neurofilament medium (NFM), a neurite specific marker, or to the presynaptic vesicle marker synaptophysin that delineates the axon terminal structure. In addition, labelled α-bungarotoxin (α-BTX), a specific nAChR antagonist, was used to delineate the end-plate (Figure 6A, B, and C). Gas7 stained in a large mass that was distinct from the staining pattern of neurofilament (NFM) and α-BTX (Figure 6A). In Gas7-deficient mice, there was far less Gas7 observed, though the distribution remained similar to the wild-type (Figure 6A). In terminal staining, Gas7 and synaptophysin partially co-localized at the central area of the presynaptic vesicle pool but not at the periphery nor at the postsynaptic region (Figure 6 B and C). The co-localization of Gas7 and synaptophysin was also seen in axon terminals in the ventral horn of the spinal cord and is consistent with our observations in NMJs (Figure S3). In previous studies, terminal Schwann cells were shown to surround the NMJ structure [38], [39], similar to Gas7 expression pattern in NMJ. Co-staining the soleus NMJ with Schwann cell marker S100 and Gas7 antibodies show that wild-type Gas7 was closely associated with the terminal Schwann cells (Figure S4A), and was distinct from the distribution of nAChR (Figure S4B–G). Comparison of the NMJ staining pattern in wild-type and Gas7-deficient mice indicates that Gas7 expression is restricted to the presynaptic nerve terminal and terminal Schwann cells, implying that it functions in the presynaptic region of the NMJ.

As shown in previous studies and verified in our model (Figure 3) [3], [5], Gas7 has been implicated in neurite outgrowth, and combined with the fact that Gas7 localized to the presynaptic region of the NMJ (Figure 6A and B), we next investigated and estimated the axon terminal branches of NMJs in wild-type Gas7 and Gas7-deficient mice. Although the number of motor neurons significantly decreased in old Gas7-deficient mice, we did not find any significant difference in the number of axon terminal branches per NMJ between the two groups by using NFM antibodies for neurite staining and α-BTX for identification of nAChR clusters (Figure 6D and E). Some reports have indicated that the remaining motor neurons will sprout new terminals (i.e., axon terminal sprouts) to reinnervate the denervated muscle fibers, if motor neurons of young adult animals are lost through aging, injury, or disease, although the new terminals are insufficient to fully compensate for the original motor units [24], [40]. Therefore, we further quantified the axon terminal sprouts at NMJs, as shown in figure 6F. About 30% of NMJs with axon terminal sprouts were detected in old Gas7-deficient mice; by contrast, the wild-type group possessed 60% of NMJs with axon terminal sprouts (Figure 6G). Thus, the Gas7-deficient mice had an impaired ability to compensate for motor neuron loss by axon terminal sprouting.

### Abnormal Muscle Fiber Subtype Composition in the Soleus of Gas7-deficient Mice

**Figure 7:**
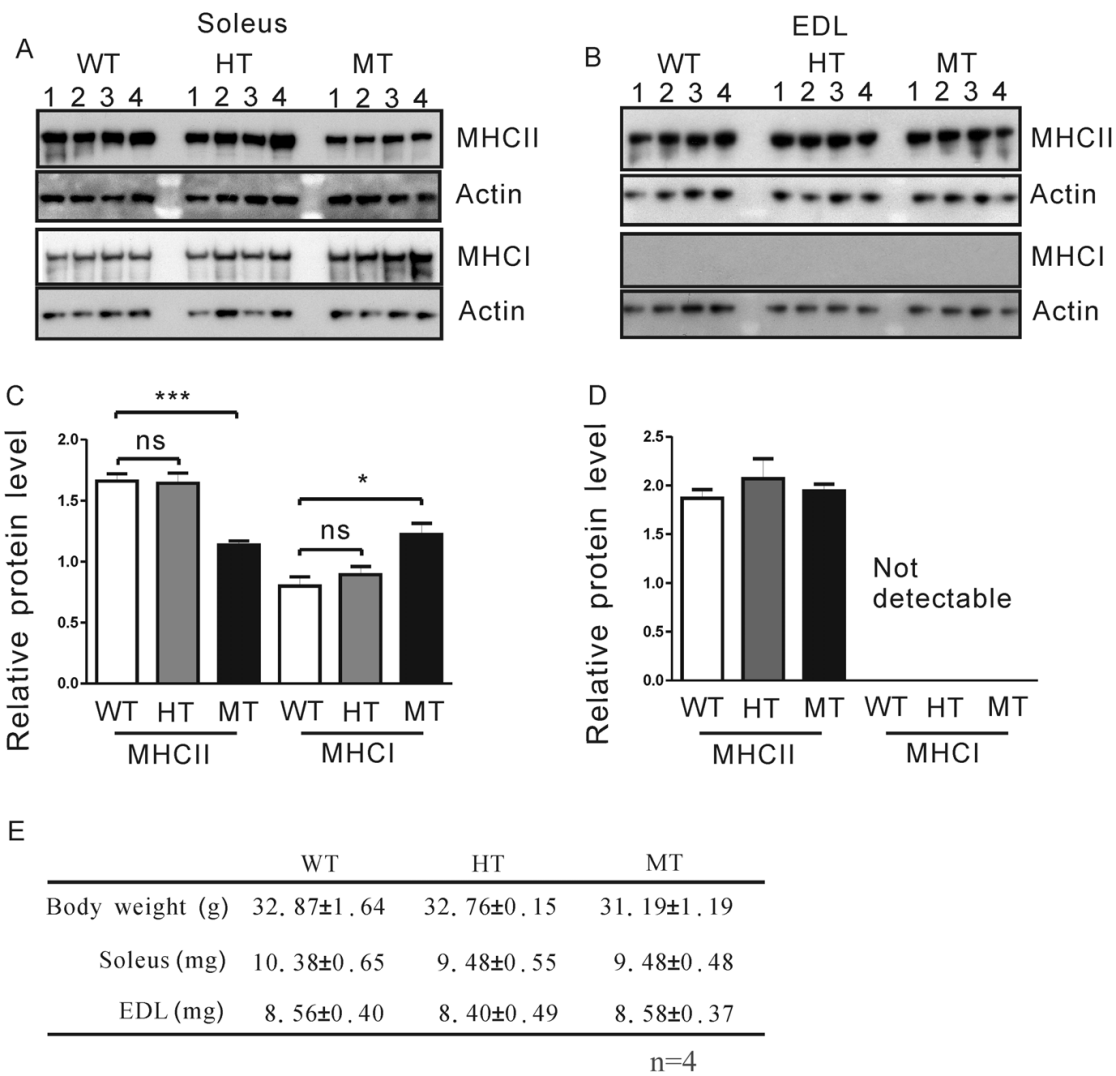
Expression of MHC II decreased in old Gas7-deficient soleus while MHC I expression increased. Myofiber subtypes in the soleus or EDL of the 12 months old wild-type and Gas7-deficient mice were determined by anti-MHC I (slow muscle fiber) and anti-MHC II (fast muscle fiber) subtype-specific antibodies. (A) Levels of MHC II expression in soleus from Gas7-deficient (MT) mice were lower than wild-type (WT) and heterozygous (HT) groups (n  = 4). (B) In EDL, no significant difference between Gas7 wild-type (WT), heterozygous (HT), and deficient (MT) mice was observed. Protein extract (2 µg), separated by NuPAGE on 3–8% gels (Invitrogen), was transferred to PVDF membranes and probed with specific antibody for MHC I (NOQ7.5.4D, Sigma) and MHC II (My32 antibody, Sigma). Internal control lanes were probed with anti-actin antibody (Chemicon) on the same membrane. (C) and (D) show histograms based on densitometry of the Western blot signals shown in (A) and (B). Levels of both types of MHC were normalized to actin (internal control) in soleus and EDL. Mean ± SEM, ***P<0.001, *P<0.05. (E) Body weight and weight of soleus and EDL muscle of Gas7 wild-type (WT), heterozygous (HT) and deficient (MT) mice.

The loss of innervation from fast α-motor neurons may for the most part contribute to the loss of fast motor units and fast fibers [16], [23]. Since old Gas7-deficient mice displayed muscle weakness and decreased axon terminal sprouts, we then asked whether muscle fiber composition is altered in these mice. We examined the soleus (containing both fast and slow muscle fibers) and extensor digitorum longus (EDL, paw extensor muscles; mainly composed of fast muscle fibers), which have been well characterized as representatives of slow or fast muscle, respectively [41]. Fast muscle fiber is recognized by antibodies against MHC II, whereas the slow muscle fiber can be detected by antibodies against MHC I [23]. Interestingly, Western blot analysis revealed lower MHC II (fast muscle fiber) and higher MHC I (slow muscle fiber) protein levels in the soleus of old homozygous Gas7-deficient mice as compared to levels in heterozygous and wild-type mice of the same age (Figure 7A and C). By contrast, we did not find any difference in the MHC II protein level in EDL muscles between animals (Figure 7B and D). Muscle samples also showed no difference in myosin isoform content between wild-type and heterozygous mice (Figure 7A and C). There were no significant differences in the total body masses or mass of the soleus or EDL muscles obtained from wild-type, heterozygous and homozygous Gas7-deficient mice (Figure 7E). Our results therefore suggest that decreased levels of Gas7 in old, homozygous mice can affect the physiology of slow muscle in the soleus but not of EDL fast muscle. On the other hand, we found that in adult mice only the level of MHC I protein was increased in the soleus of Gas7-deficient mice as compared to wild-type mice (Figure S5).

**Figure 8:**
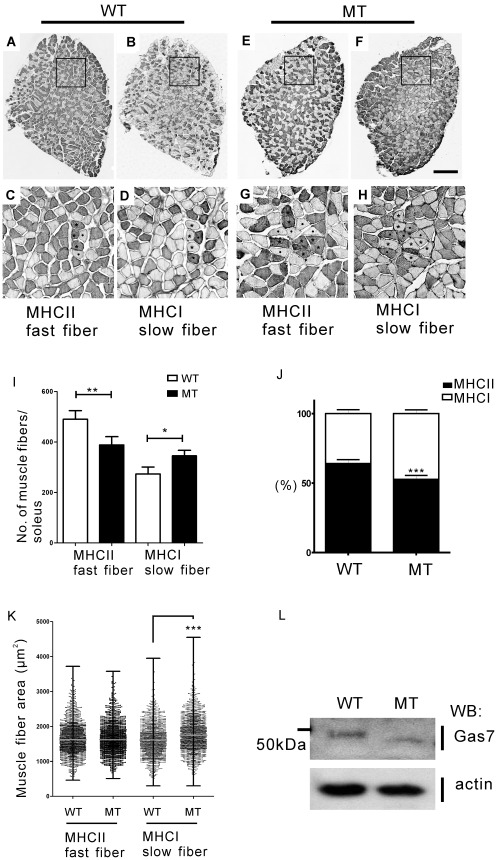
The number of fast muscle fibers decreased while the number of slow fibers increased in the soleus of aged Gas7-deficient mice as compared to wild-type mice. (A) and (B) show sequential cross-sections of 12 months old Gas7 wild-type (WT) soleus; (E) and (F) show that of Gas7-deficient (MT) soleus. Muscle fiber subtypes (slow fibers, dot; fast fibers, asterisk) were identified by staining with MHC I and MHC II antibodies (scale bar, 200 µm). Panels (C), (D), (G), and (H) show boxed areas at higher magnification. (I) Number of fibers comprising soleus. (J) Relative proportion of fast and slow fibers. (K) Measurements of the cross-sectional area of fast and slow fibers. (n  = 1500 fibers from 4 mice for each genotype). (K) The median muscle fiber area (µm^2^) with ranges containing maximum and minimum counts. (L) Western blot showing endogenous Gas7 wild-type and mutant protein expression patterns in wild-type (WT) and Gas7-deficient (MT) soleus. ***P<0.001, **P<0.01, *P<0.05.

Because the changes in the gross levels of MHC I/II isoforms pointed toward an alteration in muscle fiber subtype proportion in the soleus muscles in mutant mice, we went on to examine the cross-sectional area and the number of fast and slow muscle fibers as determined by immunohistochemical staining with MHC I and II antibodies, respectively, in the soleus and in adjacent tissue slices of old wild-type and Gas7-deficient mice (Figure 8A–D and E–H). The ratio of fast to slow muscle fibers in the soleus of wild-type Gas7 mice was about 3∶2, a ratio identical to that obtained in other studies [41]. However, the ratio of fast to slow fibers was significantly lower in Gas7-deficient mice (1∶1; Figure 8I and J). The median cross-sectional area of slow muscle fibers was slightly increased in Gas7-deficient mice, with no change seen in the fast muscle fibers (Figure 8K). In figure 8L, the expression pattern of the Gas7 mutant protein is same in the soleus as well as in the brain and spinal cord. These data are consistent with the observed variation of the protein levels of MHC isoforms and correlate with the decreased muscle strength in Gas7-deficient mice. Here, we identified defects in muscle composition, axon terminal sprouting at the NMJ, and motor neuron number in old Gas7-deficient mice, which correlate with motor activity defects seen in this group.

## Discussion

In the present study, we generated a mouse model with a labile Gas7 mutant protein that possesses the same function as wild-type Gas7. The Gas7-deficient mice have reduced motor activity and muscle strength as compared to wild-type mice. The muscle strength weakness of Gas7-deficient mice may be caused by an abnormal composition of fast and slow muscle fibers in the soleus that may correlate with a decrease in the number of motor neurons or axon terminal sprouting at NMJs during aging. Our results show that the function of Gas7 mutant protein is the same as wild-type Gas7: both proteins can rescue neurite defects caused by knockdown of endogenous Gas7 in primary cortical neurons and promote neurite outgrowth (Figure 3 and Figure S1). However, as the degradation rate of the Gas7 mutant protein is higher than that of wild-type Gas7 (Figure 2B, C and D), it appears that the stability of the Gas7 protein is critical for primary neurite length but not for primary neurite number (Figure 3). So far, the pathogenic phenotype (i.e., abnormal muscle fiber transition, decrease in motor neurons, defects in motor neuron axon terminal sprouting) exhibited in aged Gas7-deficient mice is attributed to insufficient Gas7 levels. We believe that the mouse model we generated is the first useful animal model for determining the *in vivo* function of Gas7. It is notable that the attempts of several laboratories, including ours, have been unsuccessful in generating a Gas7 knockout mouse. (e.g., http://www.knockoutmouse.org/genedetails/MGI:1202388).

Our studies on Gas7-deficient mice reveal that reduced motor neuron number is an important factor in motor activity deficiency during aging (Figure 5). However, the phenotype seems not to be severe and is only observed in aged not in adult Gas7-deficient mice. It is possible that the protein level of the labile Gas7 mutant cannot maintain the function of spinal motor neurons during aging (Figure 5A). In the case of motor neuron diseases (MND) such as ALS or SMA, loss of the survival motor neuron 1 (SMN1) gene results in severe motor activity deficiency and muscle atrophy, largely due to a massive loss of motor neurons [17], [18], [35]. In an SMA mouse model, three phenotypes were identified, and the severity of these phenotypes correlated with *smn* copy number and SMN protein level [35]. For this reason, the mild loss of motor neurons and the observed motor activity defects in Gas7-deficient mice indicates that the level of Gas7 protein may play a critical role in motor neuron function.

A deficiency in muscle fiber transition was seen in the soleus but not in EDL muscles of Gas7-deficient mice (Figures 7 and 8; Figure S5). The lack of an abnormal muscle fiber transition in EDL muscles may be explained as follows. The sequential transition between MHC isoforms is reversible from fast to slow and slow to fast: MHC I ←→ MHC IIa ←→ MHC IIx ←→ MHC IIb [42]. The soleus contains slow muscle fiber (MHC I) and fast muscle fiber (MHC IIa and IIx), while EDL is mainly composed of fast muscle fibers (MHC IIx and IIb). Thus, the abnormal transition occurring in the soleus of Gas7-deficient mice suggests that Gas7 may be involved in the transition between MHC I and MHC IIa/x isoforms.

The transformation of muscle fibers from one type to another can occur after cross-reinnervation under altered physiological conditions [43], such as during postnatal development [44], exercise [45], electrical stimulation of peripheral nerves or muscle [20], [46], or aging [47], [48]. Several studies have demonstrated that muscle fiber types can undergo transformations due to innervation or denervation by motor neurons or defects in the postsynaptic specialization [43], [49]–[51]. It has been reported that the motor neurons of young adult animals are lost during aging, and the remaining motor neurons will sprout new terminals to reinnervate the denervated muscle fibers [24]. Furthermore, age-related motor unit remodeling appears to be involved in degenerative fast muscle fibers with re-innervated axons sprouting from slow fibers [52], [53]. However, in our work, the aged Gas7-deficient mice exhibited a decreased number of motor neurons and axon terminal sprouting, but increased MHCI protein, slow muscle fiber area and ratio in the soleus (Figure 5, 6G, 7C and 8). These results suggest three possibilities that are not mutually exclusive. First, the deficiency of Gas7 may decrease axon terminal sprouting of NMJs directly. Recently, we reported that Gas7 directly enhances the activity of N-WASP to induce actin polymerization and that this activation is required for neurite outgrowth of hippocampal neurons [5]. Thus, it will be interesting to examine whether Gas7 functions with N-WASP in nerve terminal sprouting. Second, some studies indicated that the terminal Schwann cells of NMJs are critical for axon regeneration and muscle reinnervation [39], [54]. We also found that Gas7 is expressed in the terminal Schwann cells (Figure 6A and Figure S4), and, for this reason, the terminal Schwann cells with insufficient Gas7 in Gas7-deficient mice may cause axon terminal sprouting defects. Third, previous studies showed that some genes or kinases are associated with muscle contraction, metabolism, fiber transition, or gene regulation [55]–[59]. We identified Gas7 expression in wild-type skeletal muscle (soleus) and limited expression in Gas7-deficient mice (Figure 8L). Following a detailed analysis of muscle fibers, we identified strong Gas7 expression in actin and actinin stained thin filaments and MHCII expression in the thick filaments of the sarcomere in wild-type but not Gas7-deficient mice (Figure S6 and 7). The results reflect the fact that Gas7 may be involved in some muscle function autonomously. Therefore, it is difficult to conclude whether the changes in the fiber type composition and MHC isoform profiles observed in our study represent primary or secondary events. However, the results obtained from this Gas7-deficient mouse model provide a road map for further study of Gas7 function in specific tissue types.

Overall, muscle weakness in Gas7-deficient mice may be caused by a myofiber subtype transition (particularly a reduction in the proportion of fast fibers) during aging, to which possibly an inefficient motor neuron function contributes. In the present study, we have shown that Gas7 plays a physiological role in axon terminal sprouting at NMJs and muscle fiber subtype composition.

## Materials and Methods

### Ethics Statement

Mice were handled and housed in accordance with the guidelines of the National Laboratory Animal Center (NLAC) of Taiwan for Animal Care. All procedures were authorized by the Academia Sinica Institutional Animal Care and Utilization Committee (IACUC), protocol #: RMiIMBCS2009040.

### Construction and Generation of Gas7-deficient Mice

Gas7-deficient mice were generated from embryonic stem (ES) cells of the 129 mouse strain by a targeting vector containing a 1.5 kb short arm, *gas7* exon 6 b with pGK-*neo* (neomycin gene), and a 7.5 kb long arm (Figure 1A). Electroporation, selection, and screening of ES cells were conducted as reported previously [60], [61]. Selected ES clones were expanded for blastocyst injection into C57BL/6 mice to produce chimeric mice. Male chimerae with a high proportion of agouti coat color were bred with C57BL/6 females for germ line transmission of the targeted allele. The agouti heterozygous offspring were crossed to generate homozygous mutant mice which were then backcrossed for at least 7 generations with C57BL/6 mice to generate *gas7* ^+/m^ mice. These *gas7* ^+/m^ mice were interbred to generate the Gas7-deficient (*gas7* ^m/m^) mice, on an almost pure C57BL/6 genetic background. In the present study, we designated 3- to 6-month-old mice as adult mice and 12- to 15-month-old mice as old mice.

### Cell Culture and Primary Cortical Neuron Culture

HEK 293 T cells were obtained from the ATCC and grown in Dulbecco’s modified Eagle’s medium (DMEM) containing 10% fetal bovine serum, 100 ng/ml streptomycin, and 100 U/ml penicillin in humid air with 5% CO_2_ at 37°C. Primary dissected cortical neuron cells from C57BL/6 mice at embryonic day 16.5 were kept in Hanks’ balanced salt buffer with 1% glucose, 100 ng/ml streptomycin, and 100 U/ml penicillin. The tissues were digested in 0.05% trypsin with 1 mM EDTA for 3 min and triturated through a flame-polished Pasteur pipette to disrupt cell-cell adhesions. Dispersed cells were centrifuged and cultured in Neurobasal medium (Invitrogen) with B27 complex and 1 mM L-glutamine. The primary neurons were seeded on 2-well (1×10^5^ cells per well) chamber slides or a 3.5 cm^2^ dish (3×10^5^ cells per dish) coated with poly-L-lysine. The cells were cultured *in vitro* and collected for immunostaining or Western blotting. Intensities of bands of Western blotting were quantified by LAS-100 plus pictograph (Fujifilm, Tokyo, Japan).

### Plasmids Construction and Rescue Experiments

The pLKO.1-puro control and pLKO.1-puro shGas7 (TRCN0000087892) vectors were obtained from the National RNAi Core Facility, Academia Sinica, Taiwan. The Gas7 wild-type (WT) and mutant (MT) genes were cloned into pcDNA3-myc-tagged vector to obtain pcDNA3-Gas7WT-myc and MT-myc constructs. The pcDNA3-Gas7WT^ShR^-myc or pcDNA3-Gas7MT^ShR^-myc was generated by site-directed mutagenesis following the QuikChange II Site-Directed Mutagenesis Kit protocol (Stratagene, La Jolla, CA). Both clones introduced two silent mutants that while leaving the amino acid code unchanged, made Gas7 resistant to the continuously present Gas7 shRNA. The sense primer employed for the mutagenesis was: 5′-^1081^GTG GA**G** ATG AT**T** CGA CAA CAT^1101^-3′. The Gas7-specific shRNA (shGas7) targets the sequence 5′-^1081^GTG GAA ATG ATC CGA CAA CAT^1101^-3′ located on the open reading frame of *gas7.* The pLKO.1-puro control and pLKO.1-puro shGas7 vector were inserted GFP sequence (pLKO.1-puro control-GFP, pLKO.1-puro shGas7GFP) for identification of transfected cells, and packaged into lentiviral particles by the National RNAi Core Facility. To test the efficiency of the shRNA, primary cortical neurons derived from E16.5 mice were infected with the lentiviral particle. After 24 hours of infection, we transiently transfected pcDNA3-Gas7WT^ShR^-myc or pcDNA3-Gas7MT^ShR^-myc with Lipofectamine 2000 (Invitrogen, Grand Island, NY) for 4 h, then changed the medium for another 36 h of incubation. The primary neurite calculation was performed using Zeiss ZEN 2009 Lite Edition program to trace myc and βIII tubulin co-staining images (Figure S1K–N).

### Behavioral Analyses

The behavior of the animals was examined at two ages: adult mice at 3–6 months, and old mice at 12–15 months. Motor activity was tested using an accelerating rotarod treadmill (Ugo Basile, Italy). Each mouse was placed repeatedly on the rotarod until it adapted to the apparatus and could walk for 30 s at the lowest speed of 4 rpm. The rotarod was later set to accelerate from 4 to 40 rpm during a period of 5 min in each test. The latency time until a mouse was unable to walk on the rotarod was recorded with a stopwatch. Each mouse was tested twice a day (in the morning and afternoon) and monitored for 3 days. Data are presented as averages of the daily performance of all mice in a group. Hanging tests including the horizontal bar and cage wire tests were used to measure the muscle tone and strength. For the horizontal bar test, the mice were made to hang by their forelimbs from a steel rod (25 cm long, 0.3 cm in diameter), 1.5 m above a carpeted floor. The time that a mouse was able to hang on the rod was recorded, with a cut-off time of 180 s. Three trials were performed at 3-min intervals for each mouse [62], [63]. For the cage wire test, the mouse was placed on top of a wire mesh cage lid, 20 cm above the carpet. Mice were induced to grip tightly by shaking the lid lightly three times. Subsequently, the lid was turned upside-down, and the time that a mouse was able to maintain its grip on the cage wire was recorded.

### Neuromuscular Junction Immunofluorescence Staining and Confocal Image Analysis

Mice were sacrificed and evenly cardially perfused with phosphate-buffered saline (PBS) (137 mM NaCl, 2.7 mM KCl, 10 mM Na_2_HPO_4_•2H_2_O, 2 mM KH_2_PO_4_, pH7.4) followed by perfusion with 4% paraformaldehyde. Soleus muscles of Gas7 wild-type and deficient mice were quickly isolated and fixed in 4% paraformaldehyde at 4°C overnight. The whole muscle were washed 3 times in PBS for 10 minutes, then in 0.1 M sodium borohydride for 30 min to reduce the muscle background fluorescence, then twice in PBS for 30 min before immersion in blocking solution (10% normal goat serum, 1% BSA, 0.2% Triton X-100 in PBS) at 4°C overnight. The samples were then incubated at 4°C overnight with primary antibodies prepared in PBST, against Gas7 (1∶400) [3], S100 (1∶200; DAKO, Glostrup, Denmark), NFM (1∶200; Abcam, Cambridge, MA), and synaptophysin (1∶200; Chemicon, Billerica, MA). The samples were washed in PBST for 2 hours (washing solution was changed every 40 min) followed by incubation with secondary antibodies at 4°C overnight. After washing, the samples were incubated with Alexa Fluor 555-conjugated α-bungarotoxin (Invitrogen) for 2 hours, washed three times with PBST for 30 min, and then incubated in Hoechst nuclear dye (1∶500 dilution; Invitrogen) for 30 minutes. After a final wash in PBST, the samples were mounted in 30% glycerol in PBS and the NMJ morphology was examined by confocal microscopy (LSM 510, Carl Zeiss MicroImaging, Thornwood, NY).

### Analysis of Myofiber Subtypes and Gas7 Expression in Gas7 Wild-type and Deficient Mice by Western Blot

For myofiber subtype analysis, muscle samples were weighed, cut into small pieces, homogenized in RIPA buffer (50 mM Tris-HCl, pH 7.4, 1% NP-40, 150 mM NaCl, 1 mM EDTA, 1 mM phenylmethylsulfonyl fluoride, 1 mM Na_3_VO_4_, 1 mM NaF, 1 µg/ml leupeptin, 1 µg/ml aprotinin, 1 µg/ml pepstatin) using a Dounce homogenizer and centrifuged at 27,000 × *g* (4°C) for 30 minutes. Brain, spinal cord and muscle samples were prepared similarly for Gas7 expression analysis. Following protein concentration determination in supernatants obtained from muscle, brain and spinal cord samples, equal amounts of protein were separated by 3–8% NuPAGE (Invitrogen) or 10% SDS-PAGE and transferred onto polyvinylidene difluoride (PVDF) membranes (Immobilon-P; Millipore, Billerica, MA). The membrane was stained with 0.1% amido black in 5% acetic acid to confirm successful transfer of proteins. After blocking with 5% non-fat milk powder in TBS (20 mM Tris, pH 7.5, 150 mM NaCl) for 1 h at room temperature, the membrane was incubated with primary antibodies specific for Gas7 (1∶3000), myofiber subtypes II (MHC II) (My32; 1∶8,000; Sigma, St. Louis, MO) and I (MHC I slow/β cardiac) (NOQ7.5.4D, 1∶12,000; Sigma) in TBS for 1 h at room temperature. After thorough washing with TBST (TBS with 0.05% Tween 20; 3 × 15 min), the membrane was incubated with horseradish peroxidase (HRP)-conjugated secondary antibody (1∶5,000) for 1 h at room temperature. After washing, the immunoreactivity was visualized using the ECL Western Blotting Analysis System (GE Healthcare, Buckinghamshire, UK). The internal control were GAPDH, actin and α-tubulin visualized with antibody from Chemicon.

### Immunohistochemical and Immunofluoresence Staining for Spinal Motor Neuron, Muscle Fiber Number and Area, Nerve Terminus and Terminal Sprouts Calculation

Fresh spinal cord or muscle samples were fixed in 4% paraformadehyde at 4°C overnight and then transferred to 30% sucrose. After bathing in 30% sucrose at 4°C overnight, the fixed samples were embedded in OCT compound under isopentane cooled by dry ice. Subsequently, cryo-sections of the muscle samples (7 µm) or spinal cord (10 µm) were cut. For muscle fiber staining, the samples were blocked in PBS containing 10% normal goat serum, 2% BSA, and 0.2% Triton X-100 (PBST). MHC I (1∶400) and MHC II (1∶400) antibodies (Sigma) for slow and fast fiber identification were prepared in 10% normal goat serum and 1% BSA in PBST. After washing (3 × 10 min) with PBST samples were stained with secondary antibody conjugated to HRP (DAKO, Glostrup, Denmark). For motor neuron staining, the spinal cord sections were blocked by 10% normal donkey serum, 2% BSA in PBST for 1 hour and incubated with choline acetyltransferase (ChAT) (1∶200, Chemicon, Billerica, MA) antibody in PBST at 4°C overnight. After washing (3×10 min) with PBST, samples were stained with secondary antibody conjugated to Cy3 (Invitrogen). Images of cross-sections were acquired using a Zeiss LSM510 confocal microscope (Carl Zeiss Microimage, Thornwood, NY). The cross section area for muscle fibers was measured from 70–80 adjacent fibers per section taken from central regions of muscle and 4 animals per group. The areas of traced fibers were outlined and analyzed with ImageJ 1.42 software (http://rsb.info.nih.gov/ij) based on a calibrated pixel-to-actual size (µm) ratio. MetaMorph Microscopy Automation and Image Analysis Software 7.0 were used for identification and calculation of the spinal motor neurons. To visualize axon terminal sprouting in NMJ, postsynaptic AChRs were stained with Alexa Fluor 555-conjugated α-bungarotoxin (Invitrogen), and axons were stained with FITC-conjugated anti-neurofilament (NFM; Chemicon). The samples were then serially scanned under a confocal microscope (LSM 510). Obtained images were reconstructed by using Zeiss LSM image software to build z-series images. Axon termini and presynaptic vesicle pools were counted in 50 NMJs of soleus muscles isolated from adult Gas7 wild-type or deficient mice (n  = 3 per group). One motor terminal sprouting study has previously described the pattern of terminal sprouts in the NMJ [64].

### Statistical Analysis

Values presented are mean ± SEM from at least three independent experiments. The significance of differences between group means was determined by one-tailed, t-test using Prism 5 software (GraphPad Software, La Jolla, CA). A *P* value lower than 0.05 was considered statistically significant.

## Supporting Information

Figure S1 **Both wild-type and mutant Gas7 can promote neurite outgrowth.** The neurite morphology is similar in primary cortical neurons after co-transfection with plasmids carrying pLKO-GFP and pcDNA3-Gas7WT^ShR^–myc (A to D), or pcDNA3-Gas7MT^ShR^-myc (E to H). Images in (A) and (E) show staining with anti-myc antibody followed by incubation with Cy3 secondary antibody (red). (B) and (F) show the GFP expression (green). (C) and (G) show βIII-tubulin staining with anti-βIII-tubulin antibody and with polyclonal Cy5 secondary antibody (blue). (I) Statistical analysis of the average primary neurite length of cortical neurons without longest neurite. (J) There is no significant difference in the average primary neurite number per cortical neurons among GFP cotransfected with Gas7WT^ShR^ or Gas7MT^ShR^. (K) The primary neurite length calculation was based on GFP expressing primary cortical neurons with myc and βIII-tubulin staining. The confocal microscopy LSM program ZEN was used to trace myc and βIII-tubulin costaining of the neurite extending from the cell body, but does not include the longest neurite. (L-N) Box areas at high magnification show myc staining merged with βIII-tubulin staining in primary neurites. (L) Merged image with myc and βIII-tubulin signalling. (M) myc staining (red). (N) βIII-tubulin staining (blue). Scale bar, 20 µm. The data represent the mean ± SEM, ***P<0.001,*P<0.05.
(TIF)

Figure S2 **Knockdown of Gas7 did not alter brain morphology.** Despite a lack of Gas7 expression in the cortex, hippocampus, and cerebellum of 12 months old Gas7-deficient mice, the gross neuronal architecture and morphology of these brain regions were normal. Sagittal brain sections were stained with FITC-conjugated anti-Gas7 (green) and Cy3-conjugated anti-MAPII (red) antibodies. Hoechst dye for nucleus staining (blue). Matched images of the same areas in Gas7 wild-type (A-G) and Gas7-deficient mice (H-M) are shown: (A) and (H), whole brain; (B) and (I), CA1 region of the hippocampus; (C) and (J), CA2 region of the hippocampus; (D) and (K), CA3 region of hippocampus; (E) and (L), dentate gyrus region of hippocampus; (F) and (M), cortex; (G) and (M), cerebellar cortex. The lower images are magnifications of the areas outlined by the white boxes in (A) and (H).
(TIF)

Figure S3 **Gas7 is co-localized with presynaptic vesicles synapsing on alpha motor neurons in mouse spinal cord.** Gas7 was found in the spinal cord of 12 months old mouse. (A) High magnification images confirm that Gas7 co-localizes with presynaptic vesicles in synapses adjacent to motor neurons in spinal cord. A-I, FITC-conjugated anti-Gas7 (green). A-II, Cy3-conjugated synaptophysin (red). A-III, Cy5-conjugated choline acetyltransferase, a cholinergic neuron marker (ChAT, blue). A-IV, Merged image. Scale bar: 10 µm. (B) The amount of Gas7 (48 kDa) and Gas7 mutant (46 kDa) protein in cortex and spinal cord lysates of 12 months old Gas7 wild-type (WT) and deficient (MT) mice is demonstrated by a Western blot using Gas7 antibody. Cortex (Cr) sample was used as a comparable control and GAPDH was an internal control.
(TIF)

Figure S4 **Gas7 localizes to the terminal Schwann cells of the NMJ but is absent from the end-plate.** (A) Merged confocal images show that Gas7 and S100, a marker of terminal Schwann cells, co-localize; boxes A-I to A-VI showing images at high magnification. A-I, FITC-conjugated anti-Gas7 (green); A-II, Cy5-conjugated anti-S100 (red); A-III, Cy3-conjugated α-BTX (blue); A-IV, Hoechst dye for nucleus staining; A-V, superimposed image of Gas7 and S100 staining; A-VI, superimposed image of Gas7, S100, and α-BTX staining. (B to G) Side views of the presynaptic region of a NMJ to reveal the co-localization of Gas7 and S100 and its absence from the end-plate. B, FITC-conjugated anti-Gas7 (green); C, Cy5-conjugated anti-S100 (red); D, Cy3-conjugated α-BTX (blue); E, Hoechst dye for nucleus staining; F, Merged image of Gas7 and S100 staining; G, Merged image of Gas7, S100 and α-BTX staining. Scale bar represents 10 µm.
(TIF)

Figure S5 **3-month-old Gas7-deficient mice show a mild increase in MHCI protein expression in the soleus as compared with wild-type mice.** Myofiber subtypes in the soleus or EDL of the wild-type (WT) and Gas7-deficient (MT) mice in the two groups were determined by anti-MHC I (slow muscle fiber) and/or anti-MHC II (fast muscle fiber) subtype-specific antibodies. (A and C) MHC I expression in the soleus of 3 month-old Gas7-deficient (MT) mice was higher than in the wild-type (WT) (n  = 3). (B and D) In EDL, no significant difference between wild-type (WT) and Gas7-deficient (MT) mice was observed. Protein extracts (2 µg), separated on 3–8% gels (NuPAGE, Invitrogen), were transferred to PVDF membranes and probed with specific antibodies for MHC I (NOQ7.5.4D, Sigma) and MHC II (My32 antibody, Sigma). Internal control lanes were probed with anti-actin antibody on the same membrane. Levels of both types of MHC were normalized to actin (internal control) in the soleus and EDL. Data represent the mean ± SEM, *P<0.05.
(TIF)

Figure S6 **Gas7 localizes along the Z-line and thin filaments of the sarcomere in the muscle fibers.** A strong Gas7 signal was detected along the Z-line and thin filament by actinin and actin staining of the sarcomere in soleus of 12 months old Gas7 wild-type and deficient mice. (A) Low magnification view of the Gas7 expression pattern along with actinin, a marker for the Z-line of the sarcomere in muscle fibers. (B) High magnification investigation showing Gas7 merged with actinin staining. (C) Actinin staining pattern indicated by white arrows. (D) Gas7 expression surrounds the Z-line (white arrows) and possibly the M-line (asterisk) structure of sarcomere. (E) Low magnification view of Gas7 expression with actin staining of muscle fibers. (F) High magnification investigation shows that Gas7 colocalizes with actin-stained thin filament structures of the sarcomere. (G) Thin filament staining by phalloidin, an F-actin specific binding toxin. (H) Gas7 expression along the thin filament (white band). (I to L) Weak Gas7 expression in Gas7-deficient soleus stained with antibody against actinin2 (I) Low magnification view of mild Gas7 expression colocalizing with actinin in the sarcomere in Gas7-deficient muscle fibers. (J) High magnification investigation showing mild Gas7 expression colocalizing with actinin. (K) Actinin staining pattern. (L) Mild Gas7 expression in the sarcomere in Gas7-deficient muscle fibers. FITC-conjugated anti-Gas7 (green), Cy3-conjugated anti-actinin2 (red) (Abcam) for actinin staining, and Cy3-conjugated phalloidin (red) (Invitrogen) for F-actin staining. Scale bar is 10 µm.
(TIF)

Figure S7 **Gas7 colocalizes with MHCII primarily expressed in fast muscle fiber.** Confocal projections of 12 month old wild-type (A, B) and Gas7-deficient (C-J) muscle fibers of soleus were stained with Gas7 (green), MHC I (red) and II (red) antibodies. (A) Low magnification microscopy shows two kinds of Gas7 expression patterns in muscle fibers, A-a shows Gas7 merged with MHCII expression at high magnification. A-b shows another Gas7 expression pattern as supplement S6. (B) Low magnification microscopy shows that Gas7 partially colocalizes with MHCI. B-a shows Gas7 partially merged with MHCI at high magnification. B-b shows another Gas7 expression pattern as supplement S6. (C to J) Mild Gas7 expression in Gas7-deficient soleus stained with MHCII and I. FITC-conjugated anti-Gas7 (green), Cy3-conjugated anti-MHCII, a marker for fast fibers (red); Cy3-conjugated MHCI, a marker for slow fibers. Confocal images were taken by LSM 780 confocal microscope. Scale bar is 10 µm.
(TIF)

Table S1 **Body weight of adult and old Gas7 wild-type and deficient mice**
(TIF)
