# Supplementary figures and images for: Gas7-Deficient Mouse Reveals Roles in Motor Function and Muscle Fiber Composition during Aging
Source: PLoS One. 2012 May 25;7(5):e37702. doi: 10.1371/journal.pone.0037702 (PMC3360064; doi:10.1371/journal.pone.0037702)

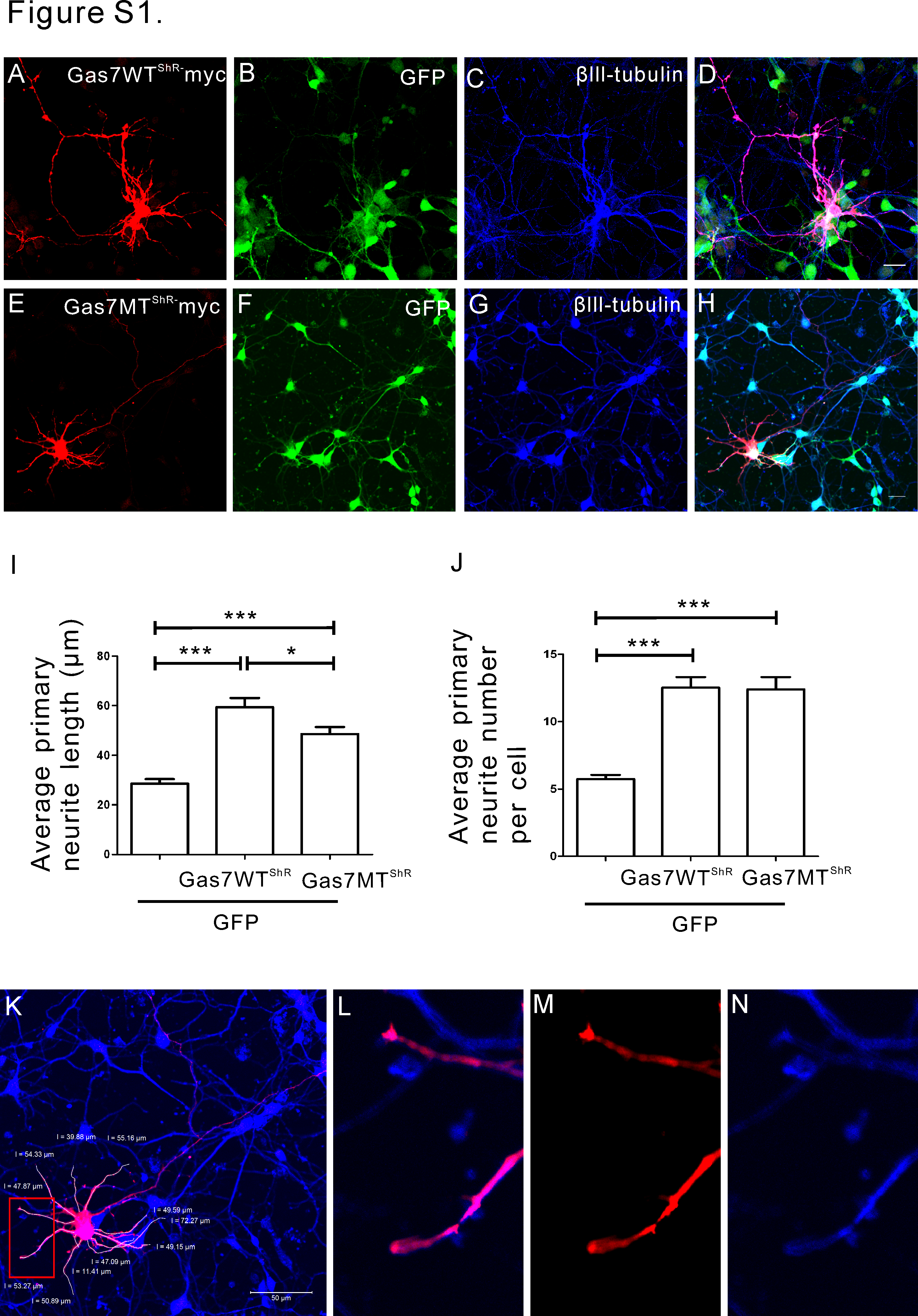

Supplement: Figure S1 — Both wild-type and mutant Gas7 can promote neurite outgrowth. The neurite morphology is similar in primary cortical neurons after co-transfection with plasmids carrying pLKO-GFP and pcDNA3-Gas7WTShR–myc (A to D), or pcDNA3-Gas7MTShR-myc (E to H). Images in (A) and (E) show staining with anti-myc antibody followed by incubation with Cy3 secondary antibody (red). (B) and (F) show the GFP expression (green). (C) and (G) show βIII-tubulin staining with anti-βIII-tubulin antibody and with polyclonal Cy5 secondary antibody (blue). (I) Statistical analysis of the average primary neurite length of cortical neurons without longest neurite. (J) There is no significant difference in the average primary neurite number per cortical neurons among GFP cotransfected with Gas7WTShR or Gas7MTShR. (K) The primary neurite length calculation was based on GFP expressing primary cortical neurons with myc and βIII-tubulin staining. The confocal microscopy LSM program ZEN was used to trace myc and βIII-tubulin costaining of the neurite extending from the cell body, but does not include the longest neurite. (L-N) Box areas at high magnification show myc staining merged with βIII-tubulin staining in primary neurites. (L) Merged image with myc and βIII-tubulin signalling. (M) myc staining (red). (N) βIII-tubulin staining (blue). Scale bar, 20 µm. The data represent the mean ± SEM, ***P<0.001,*P<0.05. (TIF) [file pone.0037702.s001.tif]

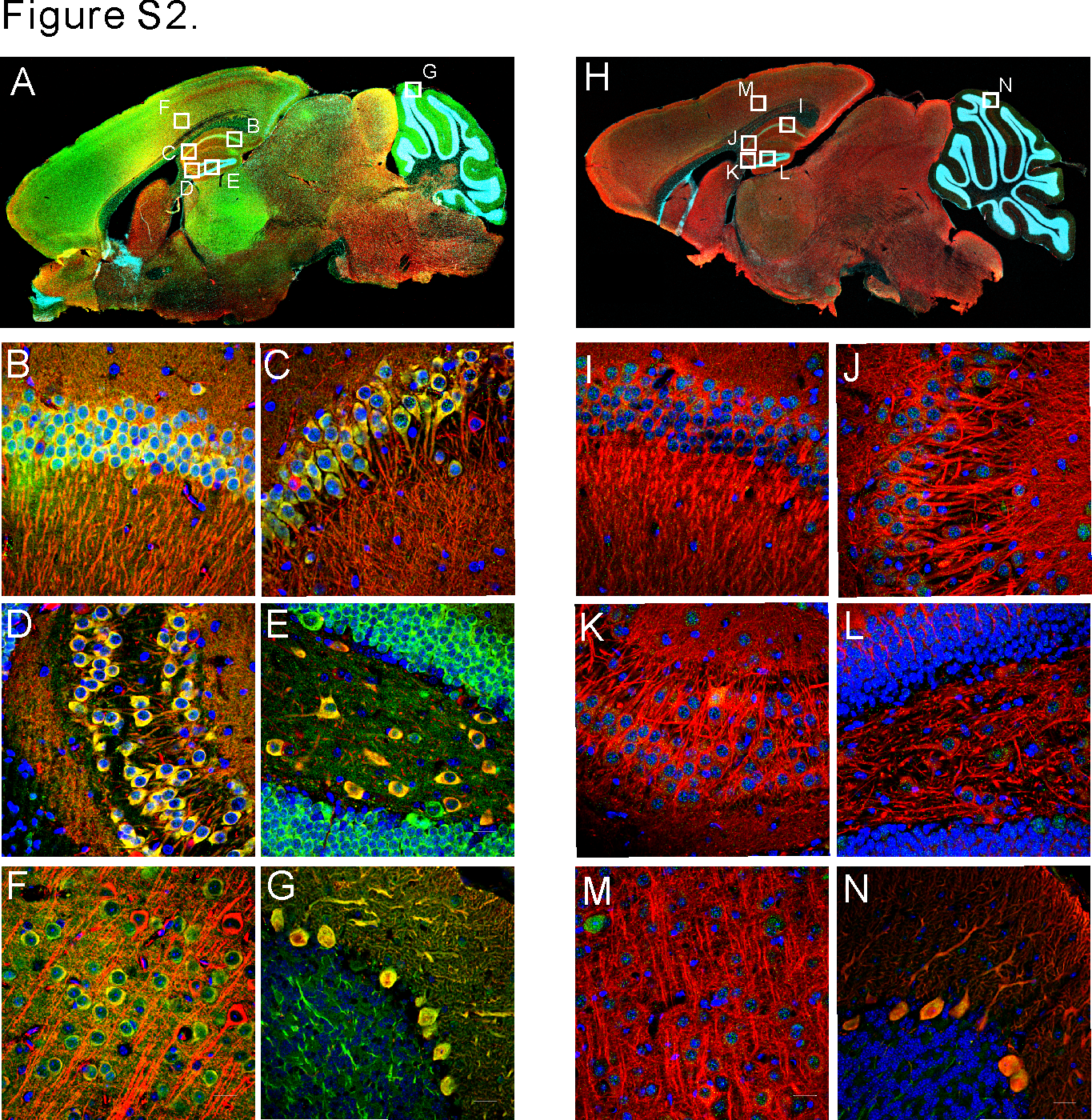

Supplement: Figure S2 — Knockdown of Gas7 did not alter brain morphology. Despite a lack of Gas7 expression in the cortex, hippocampus, and cerebellum of 12 months old Gas7-deficient mice, the gross neuronal architecture and morphology of these brain regions were normal. Sagittal brain sections were stained with FITC-conjugated anti-Gas7 (green) and Cy3-conjugated anti-MAPII (red) antibodies. Hoechst dye for nucleus staining (blue). Matched images of the same areas in Gas7 wild-type (A-G) and Gas7-deficient mice (H-M) are shown: (A) and (H), whole brain; (B) and (I), CA1 region of the hippocampus; (C) and (J), CA2 region of the hippocampus; (D) and (K), CA3 region of hippocampus; (E) and (L), dentate gyrus region of hippocampus; (F) and (M), cortex; (G) and (M), cerebellar cortex. The lower images are magnifications of the areas outlined by the white boxes in (A) and (H). (TIF) [file pone.0037702.s002.tif]

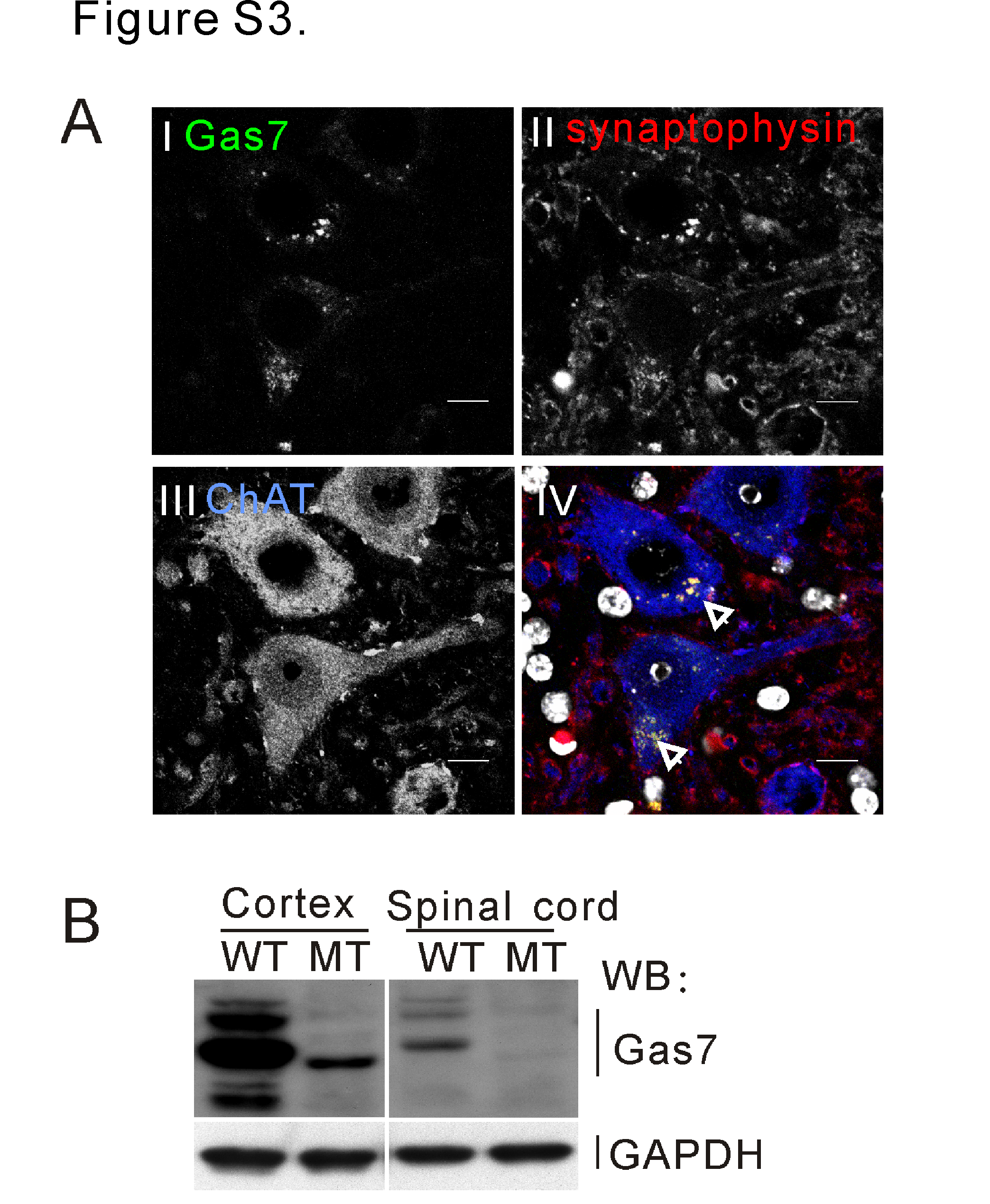

Supplement: Figure S3 — Gas7 is co-localized with presynaptic vesicles synapsing on alpha motor neurons in mouse spinal cord. Gas7 was found in the spinal cord of 12 months old mouse. (A) High magnification images confirm that Gas7 co-localizes with presynaptic vesicles in synapses adjacent to motor neurons in spinal cord. A-I, FITC-conjugated anti-Gas7 (green). A-II, Cy3-conjugated synaptophysin (red). A-III, Cy5-conjugated choline acetyltransferase, a cholinergic neuron marker (ChAT, blue). A-IV, Merged image. Scale bar: 10 µm. (B) The amount of Gas7 (48 kDa) and Gas7 mutant (46 kDa) protein in cortex and spinal cord lysates of 12 months old Gas7 wild-type (WT) and deficient (MT) mice is demonstrated by a Western blot using Gas7 antibody. Cortex (Cr) sample was used as a comparable control and GAPDH was an internal control. (TIF) [file pone.0037702.s003.tif]

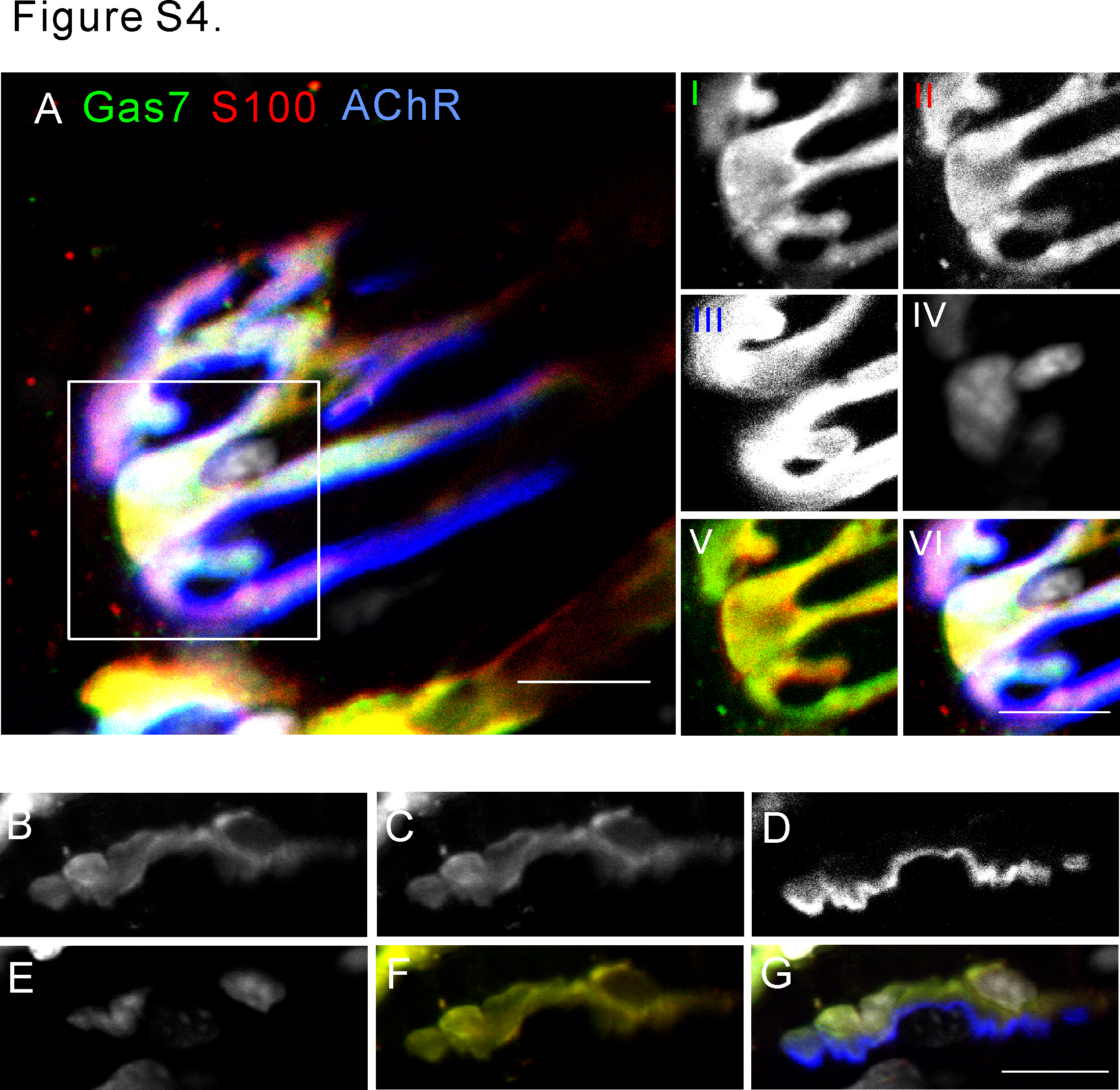

Supplement: Figure S4 — Gas7 localizes to the terminal Schwann cells of the NMJ but is absent from the end-plate. (A) Merged confocal images show that Gas7 and S100, a marker of terminal Schwann cells, co-localize; boxes A-I to A-VI showing images at high magnification. A-I, FITC-conjugated anti-Gas7 (green); A-II, Cy5-conjugated anti-S100 (red); A-III, Cy3-conjugated α-BTX (blue); A-IV, Hoechst dye for nucleus staining; A-V, superimposed image of Gas7 and S100 staining; A-VI, superimposed image of Gas7, S100, and α-BTX staining. (B to G) Side views of the presynaptic region of a NMJ to reveal the co-localization of Gas7 and S100 and its absence from the end-plate. B, FITC-conjugated anti-Gas7 (green); C, Cy5-conjugated anti-S100 (red); D, Cy3-conjugated α-BTX (blue); E, Hoechst dye for nucleus staining; F, Merged image of Gas7 and S100 staining; G, Merged image of Gas7, S100 and α-BTX staining. Scale bar represents 10 µm. (TIF) [file pone.0037702.s004.tif]

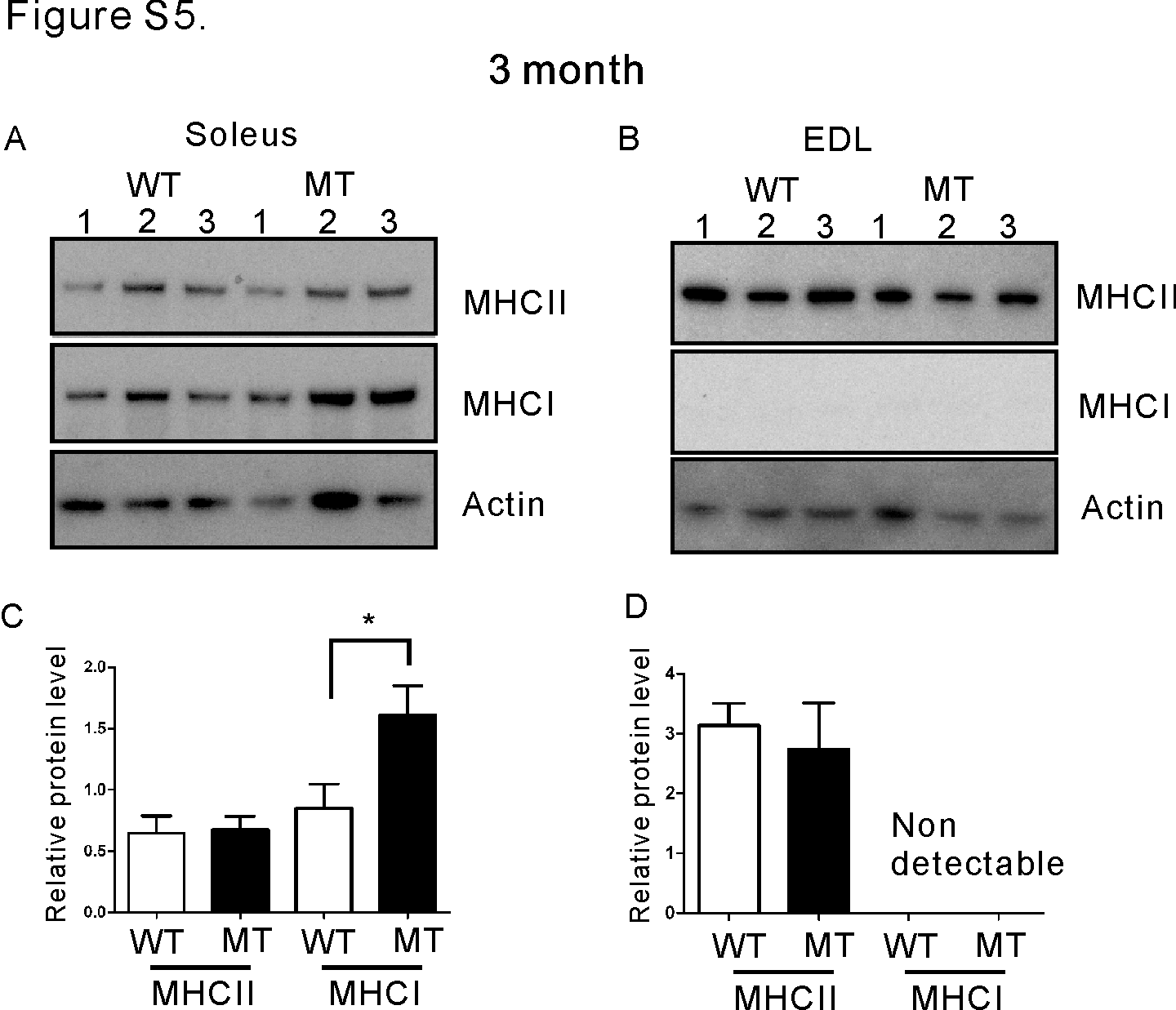

Supplement: Figure S5 — 3-month-old Gas7-deficient mice show a mild increase in MHCI protein expression in the soleus as compared with wild-type mice. Myofiber subtypes in the soleus or EDL of the wild-type (WT) and Gas7-deficient (MT) mice in the two groups were determined by anti-MHC I (slow muscle fiber) and/or anti-MHC II (fast muscle fiber) subtype-specific antibodies. (A and C) MHC I expression in the soleus of 3 month-old Gas7-deficient (MT) mice was higher than in the wild-type (WT) (n = 3). (B and D) In EDL, no significant difference between wild-type (WT) and Gas7-deficient (MT) mice was observed. Protein extracts (2 µg), separated on 3–8% gels (NuPAGE, Invitrogen), were transferred to PVDF membranes and probed with specific antibodies for MHC I (NOQ7.5.4D, Sigma) and MHC II (My32 antibody, Sigma). Internal control lanes were probed with anti-actin antibody on the same membrane. Levels of both types of MHC were normalized to actin (internal control) in the soleus and EDL. Data represent the mean ± SEM, *P<0.05. (TIF) [file pone.0037702.s005.tif]

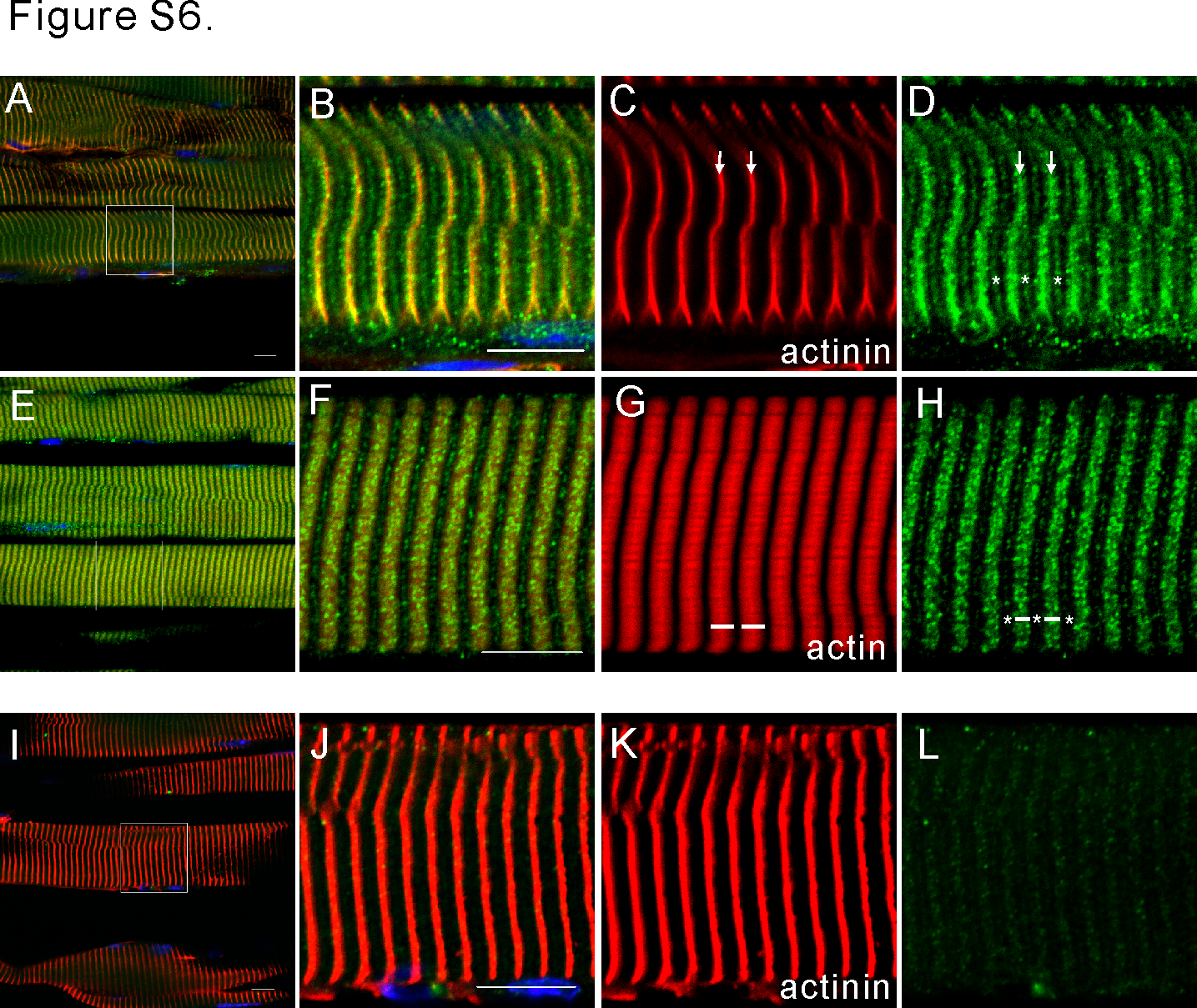

Supplement: Figure S6 — Gas7 localizes along the Z-line and thin filaments of the sarcomere in the muscle fibers. A strong Gas7 signal was detected along the Z-line and thin filament by actinin and actin staining of the sarcomere in soleus of 12 months old Gas7 wild-type and deficient mice. (A) Low magnification view of the Gas7 expression pattern along with actinin, a marker for the Z-line of the sarcomere in muscle fibers. (B) High magnification investigation showing Gas7 merged with actinin staining. (C) Actinin staining pattern indicated by white arrows. (D) Gas7 expression surrounds the Z-line (white arrows) and possibly the M-line (asterisk) structure of sarcomere. (E) Low magnification view of Gas7 expression with actin staining of muscle fibers. (F) High magnification investigation shows that Gas7 colocalizes with actin-stained thin filament structures of the sarcomere. (G) Thin filament staining by phalloidin, an F-actin specific binding toxin. (H) Gas7 expression along the thin filament (white band). (I to L) Weak Gas7 expression in Gas7-deficient soleus stained with antibody against actinin2 (I) Low magnification view of mild Gas7 expression colocalizing with actinin in the sarcomere in Gas7-deficient muscle fibers. (J) High magnification investigation showing mild Gas7 expression colocalizing with actinin. (K) Actinin staining pattern. (L) Mild Gas7 expression in the sarcomere in Gas7-deficient muscle fibers. FITC-conjugated anti-Gas7 (green), Cy3-conjugated anti-actinin2 (red) (Abcam) for actinin staining, and Cy3-conjugated phalloidin (red) (Invitrogen) for F-actin staining. Scale bar is 10 µm. (TIF) [file pone.0037702.s006.tif]

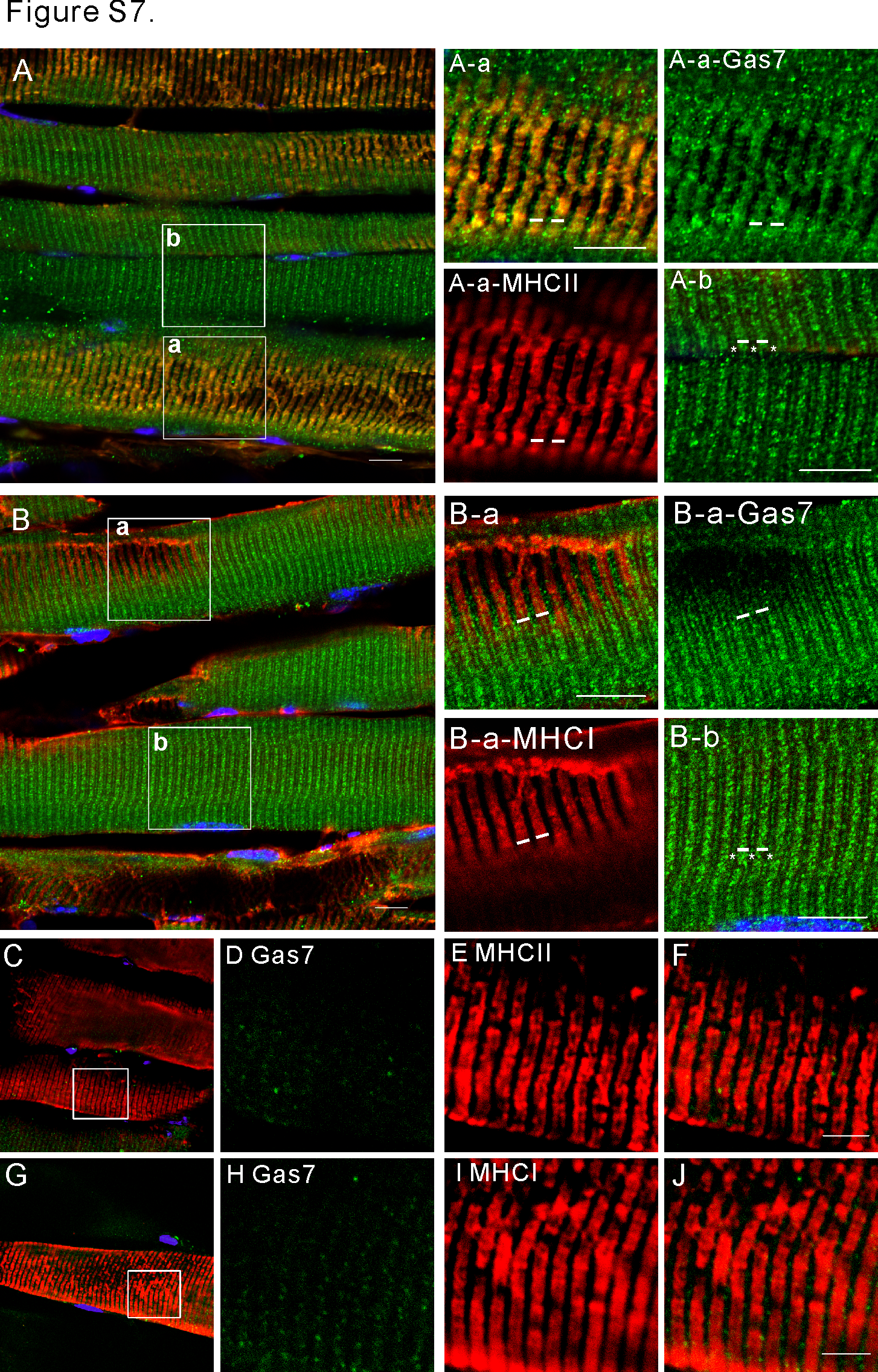

Supplement: Figure S7 — Gas7 colocalizes with MHCII primarily expressed in fast muscle fiber. Confocal projections of 12 month old wild-type (A, B) and Gas7-deficient (C-J) muscle fibers of soleus were stained with Gas7 (green), MHC I (red) and II (red) antibodies. (A) Low magnification microscopy shows two kinds of Gas7 expression patterns in muscle fibers, A-a shows Gas7 merged with MHCII expression at high magnification. A-b shows another Gas7 expression pattern as supplement S6. (B) Low magnification microscopy shows that Gas7 partially colocalizes with MHCI. B-a shows Gas7 partially merged with MHCI at high magnification. B-b shows another Gas7 expression pattern as supplement S6. (C to J) Mild Gas7 expression in Gas7-deficient soleus stained with MHCII and I. FITC-conjugated anti-Gas7 (green), Cy3-conjugated anti-MHCII, a marker for fast fibers (red); Cy3-conjugated MHCI, a marker for slow fibers. Confocal images were taken by LSM 780 confocal microscope. Scale bar is 10 µm. (TIF) [file pone.0037702.s007.tif]

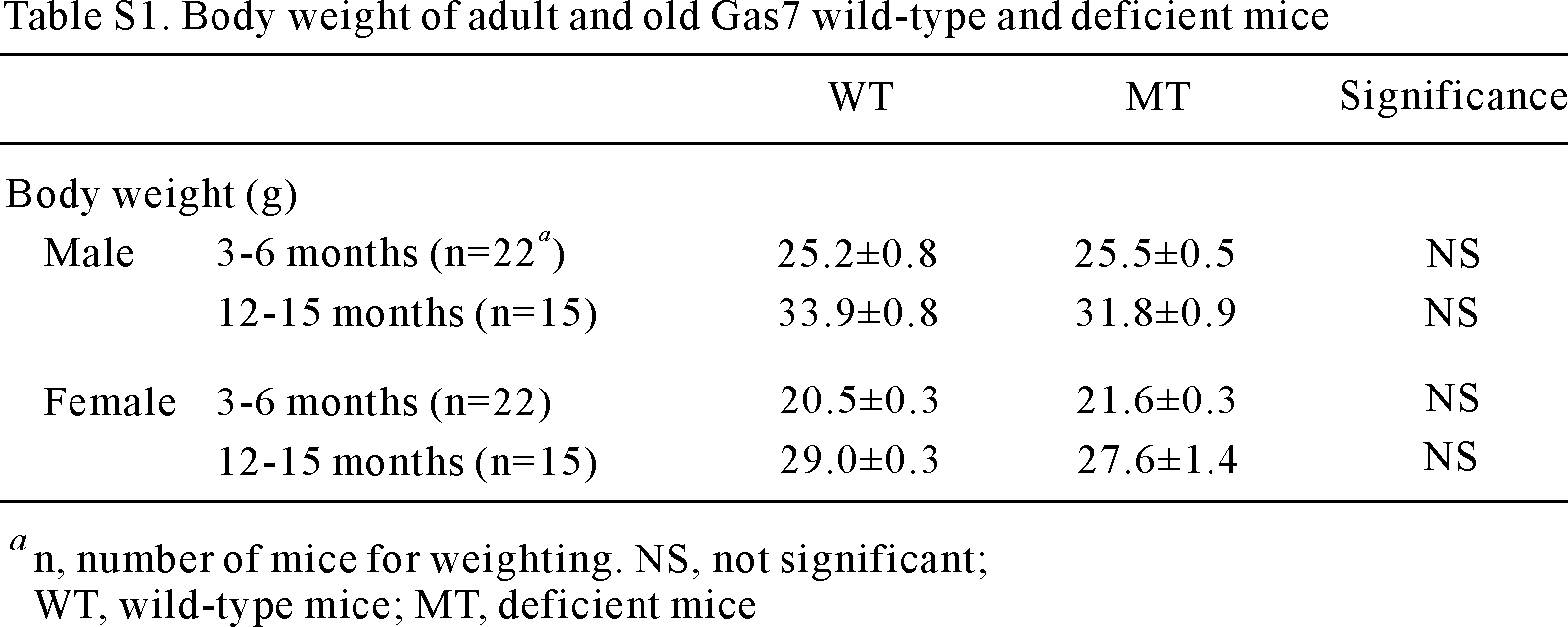

Supplement: Table S1 — Body weight of adult and old Gas7 wild-type and deficient mice (TIF) [file pone.0037702.s008.tif]
